# MiR-378a inhibits glucose metabolism by suppressing GLUT1 in prostate cancer

**DOI:** 10.1038/s41388-022-02178-0

**Published:** 2022-01-17

**Authors:** A. Cannistraci, P. Hascoet, A. Ali, P. Mundra, N. W. Clarke, V. Pavet, R. Marais

**Affiliations:** 1 Molecular Oncology Group, Cancer Research UK Manchester Institute, The University of Manchester, Alderley Park, Macclesfield, Cheshire, SK10 4TG UK; 2 Genito-Urinary Cancer Research Group and the FASTMAN Prostate Cancer Centre for Excellence, Division of Cancer Sciences, Manchester Cancer Research Centre, The University of Manchester, 555 Wilmslow Road, Manchester, M20 4GJ UK; 3 The Christie NHS Foundation Trust, Wilmslow Road, Manchester, M20 4BX UK

**Keywords:** Cancer metabolism, Tumour biomarkers

## Abstract

Prostate cancer (PCa) is the fifth leading cause of cancer related deaths worldwide, in part due to a lack of molecular stratification tools that can distinguish primary tumours that will remain indolent from those that will metastasise. Amongst potential molecular biomarkers, microRNAs (miRs) have attracted particular interest because of their high stability in body fluids and fixed tissues. These small non-coding RNAs modulate several physiological and pathological processes, including cancer progression. Herein we explore the prognostic potential and the functional role of miRs in localised PCa and their relation to nodal metastasis. We define a 7-miR signature that is associated with poor survival independently of age, Gleason score, pathological T state, N stage and surgical margin status and that is also prognostic for disease-free survival in patients with intermediate-risk localised disease. Within our 7-miR signature, we show that miR-378a-3p (hereafter miR-378a) levels are low in primary tumours compared to benign prostate tissue, and also lower in Gleason score 8–9 compared to Gleason 6–7 PCa. We demonstrate that miR-378a impairs glucose metabolism and reduces proliferation in PCa cells through independent mechanisms, and we identify glucose transporter 1 (GLUT1) messenger RNA as a direct target of miR-378a. We show that GLUT1 inhibition hampers glycolysis, leading to cell death. Our data provides a rational for a new PCa stratification strategy based on miR expression, and it reveals that miR-378a and GLUT1 are potential therapeutic targets in highly aggressive glycolytic PCa.

## Introduction

Prostate cancer (PCa) is the second most common cancer in men [1] and its detection and reported incidence have increased over the past two decades, in part due to widespread PSA testing, particularly in the developed world. While early detection allows therapeutic intervention before metastasis, it also causes widespread concerns as overtreatment can lead to unnecessary morbidities, a position made worse by the lack of effective tools to distinguish indolent from aggressive disease. Current efforts to develop biomarkers for risk stratification in PCa include assessment of DNA and RNA indices [2, 3], but the RNA indices largely exclude microRNAs (miRs), highly conserved small (21–23nt) non-coding RNA molecules that regulate many cellular processes in response to endogenous and exogenous stimuli [4].

MiRs function to fine-tune cell behaviour by modulating rapid post-transcriptional changes in cellular proteomes by degrading target mRNAs, or inhibiting their translation; notably, miR expression patterns are altered in many diseases including PCa [5, 6]. Being highly stable in body fluids and fixed tissues, miR are attractive biomarkers of prognosis and response to therapy and ideal candidates for evaluating tumour pathophysiology [7]. Herein, we analyse miR expression in primary PCa and identify seven fully processed miRs associated with nodal metastasis. We demonstrate that these miRs can segregate patients with localised PCa into groups with different risk of disease progression, independent of age, Gleason score, pathological T state, N stage and surgical margin status. Our 7-miR signature is also prognostic for disease-free survival in patients with intermediate-risk localised disease. Furthermore, we show that low expression of miR-378a, a member of our 7-miR signature, is important for cell cycle progression and homeostatic cellular glycolysis, as a rise in miR-378a levels results in G1/S cell cycle arrest and altered metabolism in PCa cells. Interestingly, we found that miR-378a blocks glycolysis by direct targeting of the Glucose Transporter 1/Solute Carrier 2A1 (GLUT1/SLC2A1; hereafter GLUT1) in PCa cells, ultimately resulting in cell death. Together, we define a 7-miR signature that is prognostic in patients where clinical decisions must balance the uncertainty of progression against treatments that could cause severe morbidity without benefit, and we show a key role of miR-378a in regulating PCa metabolism, allowing us to identify GLUT1 as a therapeutic target in early stage PCa with glycolytic features.

## Results

### A prognostic 7-miR signature in prostate cancer

Prostate cancer patients presenting with lymph node metastasis (N1) have a high risk of recurrence after radical prostatectomy and an increased mortality rate compared with patients with localised disease (N0) [8]. Previous studies report the presence of metastatic subclones within original primary lesions [9, 10], so we reasoned that the molecular mechanisms underpinning metastasis in N1 patients could be traced back to the primary tumour. Given the potential of miRs as orchestrators of disease onset and progression, we examined whether the presence of specific miRs in primary tumours could identify which patients would develop metastasis. To that aim, we examined RNAseq data from primary PCa in The Cancer Genome Atlas (TCGA) repository [11–14] and compared miR expression in N0 and N1 PCa (Supplementary Table 1a) [15, 16].

Of the ten molecules showing the highest differential expression (Supplementary Table 2), seven miRs were detected in more than 99% of the patients as fully processed molecules with the potential to regulate biological functions. Unsupervised hierarchical clustering based on the expression of these 7 miRs stratified the TCGA patients into two groups, Group 1 and Group 2 (Supplementary Fig. 1a and Supplementary Table 1a). Group 1 was enriched for N1 patients and group 2 was enriched for N0 patients (Supplementary Fig. 1b). Group 1 had statistically significant poorer disease-free survival (DFS) than Group 2 (Supplementary Fig. 1c), and multivariate Cox regression analysis showed that this was independent of stage (N0 vs N1), age, Gleason score (≤7 vs 8–10), pathological T stage (pT2 vs pT3/pT4) and surgical margin status (negative vs positive) (HR = 1.96, 95% CI 1.11–3.46, *p* = 0.020).

**Fig. 1:**
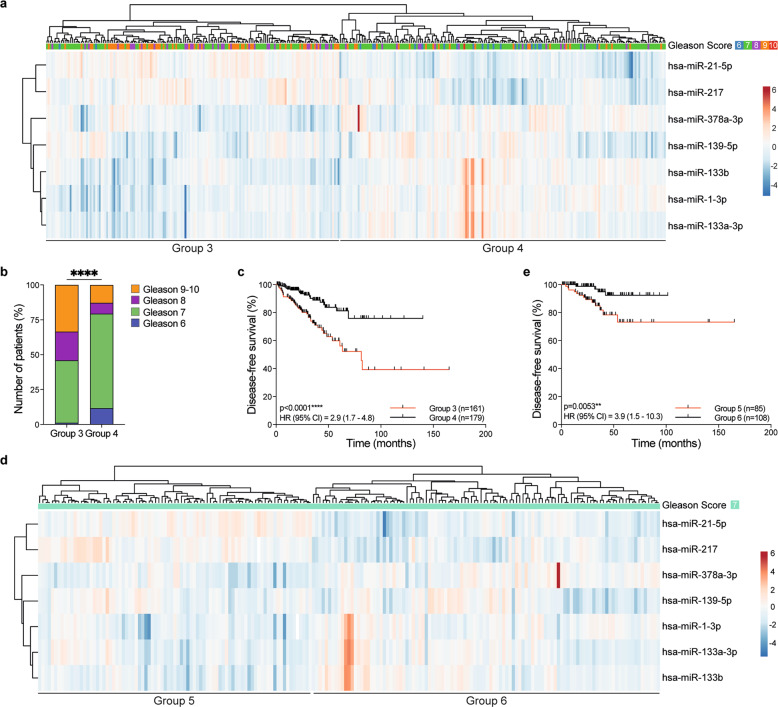
A 7-miRs prognostic signature in primary human prostate cancer. **a** Unsupervised hierarchical clustering of TCGA N0 PCa patients using expression (log2 RPM [reads per million]) of 7-miRs identified from miR RNAseq data. Columns: individual patients, rows: individual miRs. Centering and unit variance scaling are applied to rows, and rows and columns are clustered using correlation distance and average linkage. The dendrogram at the top shows Gleason scores (G6: blue; G7: green; G8: purple; G9: orange; G10: red). **b** Bar chart showing the Gleason score distribution among patients (*y*-axis) in Groups 3 and 4 (*x*-axis). G6: blue; G7: green; G8: purple; G9-10: orange. ****p ≤ 0.0001; Chi-square test. **c** Kaplan–Meier plot of disease-free survival in Groups 3 and 4 patients. The number of patients (*n*) in each group is indicated. ****p ≤ 0.0001; 95% CI = 2.9 (1.7–4.8); Mantel–Cox test. Median survival Group 3: 81.24 months; median survival Group 4: not reached. **d** Unsupervised hierarchical clustering analysis of TCGA N0 PCa patients presenting Gleason 7 score using expression (log2 RPM) of 7-miR identified from miR RNAseq data. Columns: individual patients, rows: individual miRs. Centering and unit variance scaling are applied to rows, and rows and columns are clustered using correlation distance and average linkage. The dendrogram shows Gleason score for each patient (G7: green). **e** Kaplan–Meier plot of disease-free survival in Group 5 and Group 6 patients. The number of patients (*n*) in each group is indicated. **p = 0.0053; HR 95% CI = 3.9 (1.5–10.3); Mantel–Cox test. Median survival Group 5: not reached; median survival Group 6: not reached.

Thus, we defined a 7-miR signature that was prognostic for outcome in PCa. Notably, within the N0 patients, our 7-miR signature also distinguished two prognostic groups, Group 3 and Group 4 (Fig. 1a; Supplementary Table 1a). Group 3 was enriched for patients with Gleason score 8–9, whereas Group 4 was enriched for patients with Gleason score 6–7 (Fig. 1b) and the Group 4 patients had significantly better DFS than the Group 3 patients (Fig. 1c), independent of age, Gleason score (≤7 vs 8–10), pathological T stage (pT2 vs pT3/pT4), and surgical margin status (negative vs positive) (multivariate Cox regression analysis; HR = 2.00 (1.09–3.67), *p* = 0.026). Finally, our 7-miR signature also defined two prognostic groups within patients with Gleason score 7 patients, Group 5 and Group 6 (Fig. 1d), with Group 6 having significantly better DFS than Group 5 (Fig. 1e), independent of age, pathological T stage (pT2 vs pT3/pT4) and surgical margin status (negative vs. positive) (multivariate Cox regression analysis; HR = 4.83 (1.55–15.06), *p* = 0.007).

**Fig. 2:**
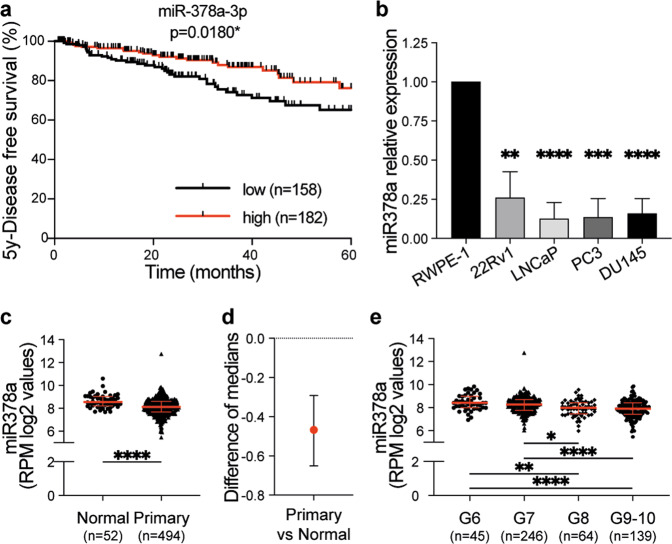
MiR-378a expression is reduced in advanced prostate cancer. **a** Kaplan–Meier plot showing 5-years disease-free survival of TCGA PCa patients with localised disease (N0) grouped according to miR-378a-3p expression (log2 RPM). The average expression at baseline was used to assign patients to “low” (below average) or “high” (above average) subgroups. The number of patients (*n*) in each group is indicated. *p = 0.018; 95% CI = 1.9 (1.1–3.2); Mantel–Cox test. **b** Graph showing *miR-378a* expression normalised to *Rnu6b* in 22Rv1, LNCaP, PC3 and DU145 PCa cell lines relative to RWPE-1 cells (fold from comparative Ct of RT-qPCR). Data are from three independent biological replicates each performed in technical duplicates and show mean ± SD; **p ≤ 0.01, ***p ≤ 0.001, ****p ≤ 0.0001; unpaired Student’s *t* test. **c** Graph showing *miR-378a* expression (log2 RPM) in normal prostate and primary prostate tumour from the TCGA dataset. Data is displayed as the median with the interquartile range. ****p ≤ 0.0001; 95% CI; unpaired two-tailed Mann–Whitney test. **d** Graph showing the difference of medians between the primary tumours and the normal tissues from the TCGA dataset. ****p ≤ 0.0001; 95% CI; unpaired two-tailed Mann–Whitney test. **e** Graph showing *miR-378a* expression (log2 RPM) in primary prostate tumour from the TCGA dataset according to Gleason score (G6: Gleason 6; G7: Gleason 7; G8: Gleason 8; G9–10: Gleason 9 and Gleason 10). Data is displayed as the median with the interquartile range. Comparisons between multiple groups were performed by applying a non-parametric Kruskal–Wallis test with Dunn’s correction for multiple comparisons (significance level of 0.05; *adjusted *p* ≤ 0.05, **adjusted *p* ≤ 0.01,****adjusted *p* ≤ 0.0001).

Note that the individual expression levels of six of the miRs in our signature correlated with prognosis (*p* value ≤ 0.05), with high expression of miR-21–5p or miR-217, or low expression of miR-1-3p, miR-133b, miR-133a-3p or miR-378a-3p (hereafter miR-378a) correlating with poorer DFS in the N0 patients (Fig. 2a; Supplementary Fig. 2a–f). Amongst these miRs, miR-1-3p and miR-133b were prognostic independent of age, Gleason score (≤ 7 vs 8–10), pathological T stage (pT2 vs pT3/pT4), and surgical margin status (negative vs positive) (multivariate Cox regression analysis; miR-1-3p: HR = 1.81 (1.03–3.17), *p* = 0.040; miR-133b: HR = 1.86 (1.09–3.19), *p* = 0.023).

### miR-378a levels are low in prostate cancer

Taken together, our data above revealed a 7-miR signature that was prognostic for outcome in PCa and which distinguishes N0 patients with a higher likelihood of progression, supporting our hypothesis that the molecular features of metastasis can be traced back to the primary tumour. Importantly, this signature also identified which Gleason 7 patients were likely to progress, an important advance, because these patients are clinically challenging as their disease can follow very different clinical courses [17]. Of the six mature miRs whose expression correlated with patient survival, only miR-21-5p and miR-378a showed robust expression in a panel of PCa cells that included immortalised normal epithelial cells (RWPE-1), primary prostate adenocarcinoma cells (22Rv1) and metastatic PCa cells from lymph node (LNCaP), bone (PC3) and brain (DU145) (Supplementary Tables 3 and 4). Previous studies have established that miR-21-5p is associated with increased PCa metastasis [18–22] but little is known about the role of miR-378a. We showed that miR-378a levels were significantly lower in 22Rv1, LNCaP, PC3 and DU145 cancer cells than in RWPE-1 cells (Fig. 2b). We also demonstrated that miR-378a expression was significantly lower in LNCaP and PC3 cells than in immortalised benign prostate hyperplasia BPH-1 cells (Supplementary Fig. 3a). We determined that miR-378a expression was reduced in primary prostate tumours compared to benign prostate tissue in the TCGA and Memorial Sloan-Kettering Cancer Center (MSKCC) [23] datasets (Fig. 2c, d, Supplementary Fig. 3b, c and Supplementary Table 1b) but not between primary and metastatic samples in the MSKCC cohort (Supplementary Fig. 3b). Finally, miR-378a levels were lower in Gleason 8 than in Gleason 6/7 tumours (Adj. *p* value = 0.01) (Fig. 2e, Supplementary Fig. 3d). Thus, we identified an inverse correlation between miR-378a expression and early disease progression in PCa.

### MiR-378a inhibits proliferation and glucose metabolism in prostate cells

**Fig. 3:**
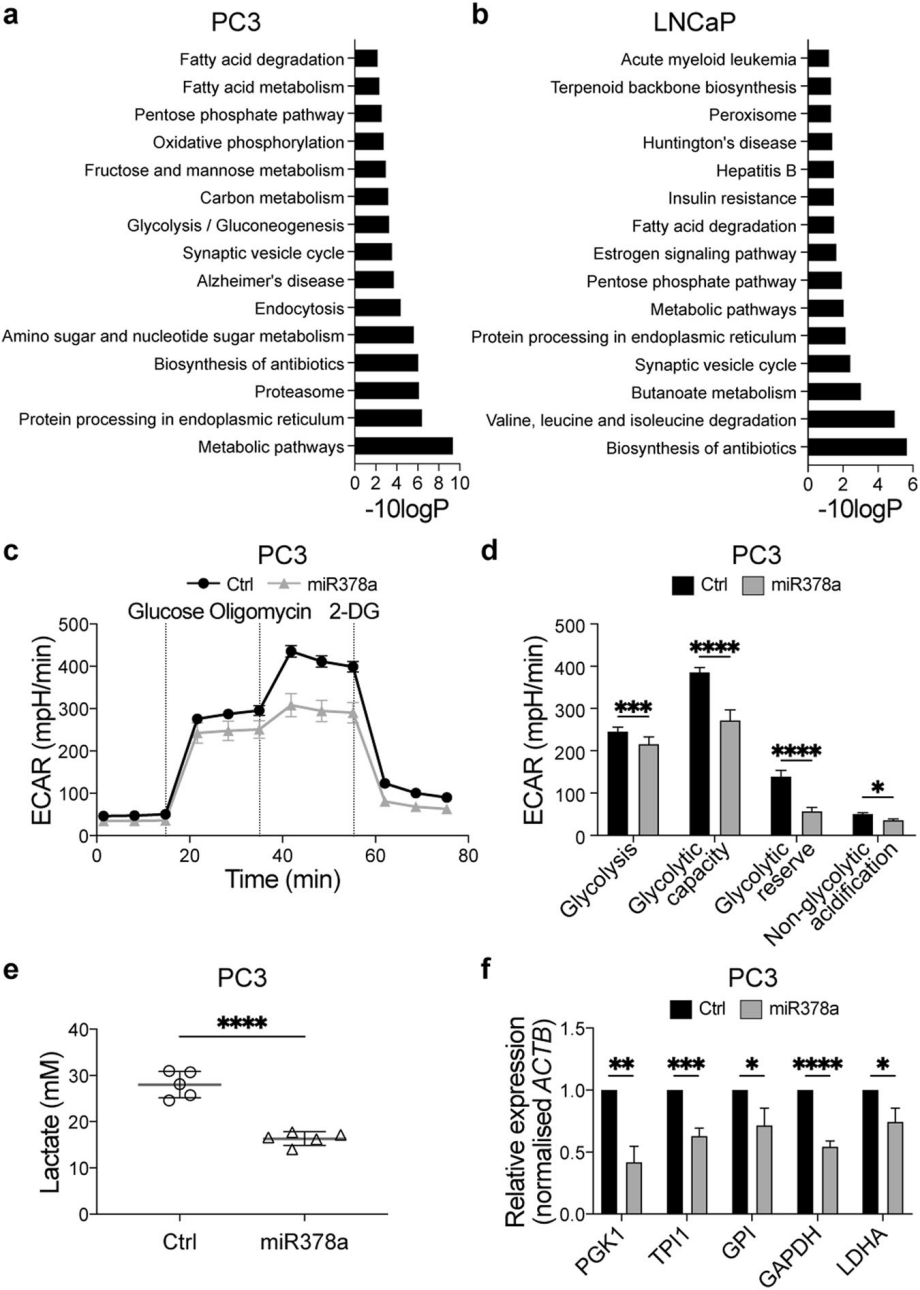
MiR-378a regulates prostate cancer cell metabolism. Top 15 deregulated pathways in PC3 cells (**a**) and LNCaP cells (**b**) from TMT-Mass spectrometry. Analysis was performed at 72 h after transfection with a miR-378a-mimic or a non-targeting control. Differentially expressed proteins were filtered using a cut-off of “unique-peptides/number-of-peptides” ratio ≥0.25 and a PEAKS significance value ≥10. Pathway analysis was performed with the David Functional annotation tool (default settings used - version 6.8) and the KEGG dataset. **c** Graph showing extracellular acidification rate (ECAR) in PC3 cells 72 h after transfection with a miR-378a-mimic or non-targeting control (Ctrl) and challenged with glucose (10 mM), oligomycin (1 µM) and 2-DG (50 mM). Values are normalised to total protein content. **d** Quantification of the glyco-stress test parameters in PC3 cells from the normalised ECAR values from (**c**). One representative experiment out of three independent biological replicates with ten technical replicates each is shown as mean ± SD; *adjusted *p* ≤ 0.05, ****adjusted *p* ≤ 0.0001; unpaired parametric Student’s *t* test using the Holm–Sidak method to correct for multiple comparisons (α = 0.05). **e** Quantification of extracellular lactate in PC3 cells 72 h after transfection with a miR-378a-mimic or non-targeting control (Ctrl). Data are from one experiment with five technical replicates and show mean ± SD; ****p ≤ 0.0001; unpaired two-tailed Student’s *t* test. **f** Graph showing expression of mRNAs encoding glycolysis rate limiting enzymes normalised to *Actb* (fold from comparative Ct values) in PC3 cells 72 h after transfection with a miR-378a-mimic or non-targeting control (Ctrl). Data are from three independent biological replicates each performed in technical duplicates and show mean ± SD; *p ≤ 0.05; **p ≤ 0.01; ***p ≤ 0.001; ****p ≤ 0.0001; unpaired two-tailed Student’s *t* test.

The reduced expression of miR-378a in primary tumours suggests that it may regulate the levels of mRNAs or proteins involved in disease progression. We performed RNAseq in PC3 and LNCaP cells transfected with a miR-378a mimic or a non-targeting miR control, and found significant changes in genes related to glucose metabolism, including glycolysis, the pentose phosphate pathway and oxidative phosphorylation in PC3 cells, and fatty acid metabolism and starch and sucrose signalling in LNCaP cells (Supplementary Fig. 4a). Note that cells transfected with miR-378a showed an alteration in the Hallmark glycolysis (46/200 genes of this gene set) in both LNCaP and PC3 cells, 40 in the same direction (Supplementary Fig. 4b). Thus, to further investigate how miR-378a affected the proteomes of these cells and further confirm its impact on metabolic pathways, we performed isobaric-tagged quantitative mass-spectrometry in PC3 and LNCaP cells transfected with the miR-378a mimic or a non-targeting control [24]. Notably, several metabolic cascades, including the glycolysis/gluconeogenesis, pentose phosphate pathway and fatty acid metabolism were altered amongst others in PC3 and/or LNCaP cells upon miR-378a transfection (Fig. 3a, b). Since these processes are regulated by the availability of the metabolites of glucose catabolism, our data suggested that miR-378a regulated glycolysis in these cells, an intriguing observation because increased metabolic activity through this pathway is associated with more aggressive PCa [25–27].

To investigate how miR-378a regulated PCa glycolysis, we transfected PC3 and LNCaP cells with the miR-378a mimic or non-targeting control and measured the extracellular acidification rate (ECAR) of cells exposed to glucose-free medium and then sequentially challenged with glucose, the ATP synthetase/mitochondrial respiration inhibitor oligomycin, and the glucose metabolism inhibitor 2-deoxyglucose (2-DG). Compared to the non-targeting control, miR-378a reduced ECAR when both cell lines were challenged with glucose, oligomycin and 2-DG (Fig. 3c, d; Supplementary Fig. 4c, d). Accordingly, miR-378a induced a significant reduction in extracellular lactate in PC3 and LNCaP cells (Fig. 3e; Supplementary Fig. 4e), and a significant decrease in the key rate-limiting glycolytic enzymes *PGK1*, *TPI1*, *GPI*, *GAPDH* and *LDHA* in PC3 cells (Fig. 3f).

Thus, miR-378a suppressed the ability of PCa cells to metabolise glucose through the glycolytic pathway. Notably, in other cancers, suppression of glycolysis triggered a metabolic switch that upregulated mitochondrial function and enhanced oxidative phosphorylation to provide the energy needed for survival [28, 29]. However, our data showed that in PC3 and LNCaP cells, miR-378a inhibited glycolysis without affecting oxygen consumption (Supplementary Fig. 5a–d). Thus, unlike other cancers, when glycolysis was inhibited by miR-378a, mitochondrial respiration did not counterbalance the energy requirements of PCa cells.

It was previously reported that miR-378a inhibited PCa cell proliferation [30]. Accordingly, we observed that the miR-378a mimic downregulated CCND1 and upregulated P27 in PC3 and LNCaP cells (Supplementary Fig. 6a). This correlated with a G1/S cell cycle arrest and an accumulation of cells in G0/G1, reduced proliferation and decreased colony formation (Supplementary Fig. 6b–f). Critically, when we induced a G1/S cell cycle arrest in PC3 and LNCaP cells using the CDK4/CDK6 inhibitor abemaciclib (Supplementary Fig. 7a–d), we did not observe decreased glycolysis of these cells (Supplementary Fig. 7e, f). Together these results indicated that the regulation of glycolysis by miR-378a was independent of cell cycle in these PCa cells, suggesting that miR-378a regulated glycolysis and proliferation through independent mechanisms.

### MiR-378a regulates GLUT1 and WBP2 expression in PCa

**Fig. 4:**
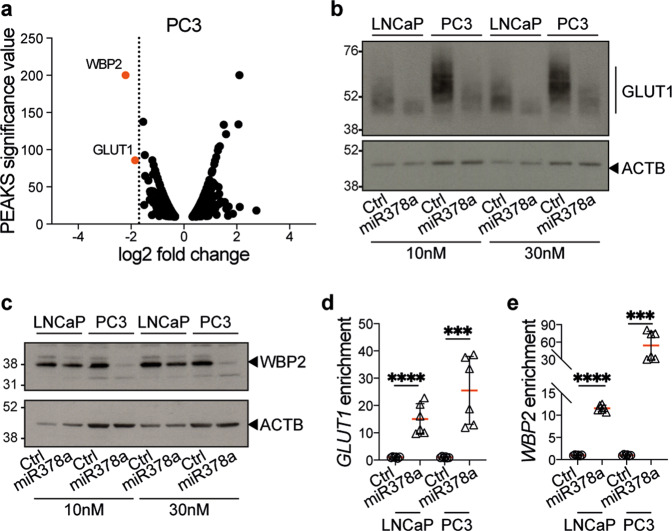
MiR-378a downregulates GLUT1 and WBP2 by direct binding to their transcripts. **a** Volcano plot showing significant changes (fold change) in proteins identified by mass-spectrometry in PC3 cells at 72 h after transfection with the miR-378a-mimic compared to a non-targeting control. Differentially expressed proteins were filtered using a cut-off of “unique-peptides/number-of-peptides” ratio ≥0.25 and a PEAKS significance value ≥10. Orange dots highlight the most significant downregulated proteins (WBP2, GLUT1). **b** Western blot for GLUT1 and ACTB as loading control in PC3 and LNCaP cells at 72 h after transfection with increasing concentrations (10 nM and 30 nM) of the miR-378a-mimic or a non-targeting control (Ctrl). **c** Western blot for WBP2 and ACTB as loading control in PC3 and LNCaP cells at 72 h after transfection with increasing concentrations (10 nM and 30 nM) of the miR-378a-mimic or a non-targeting control (Ctrl). Graphs showing *GLUT1* (**d**) and *WBP2* (**e**) mRNA levels normalised over input from miR-mRNA capture assays of PC3 or LNCaP cells at 24 h after transfection with a biotinylated miR-378a-mimic or a biotinylated non-targeting control (Ctrl). Data is the mean ± SD of two independent biological replicates, each performed as technical triplicates; ***p ≤ 0.001, ****p ≤ 0.0001; unpaired two-tailed Student’s *t* test.

MiRs exert their biological effects by regulating protein levels, either by impairing protein translation and/or by inducing target mRNA degradation [31]. Our quantitative mass-spectrometry revealed that the WW domain-binding protein 2 (WBP2) and the glucose transporter GLUT1 were the most downregulated proteins in PC3 cells transfected with the miR-378a mimic (Fig. 4a; Supplementary Fig. 8a, b). Moreover, GLUT1 but not WBP2 was also reduced in LNCaP cells, albeit to a lesser extent (Supplementary Fig. 8c–e). We confirmed by western blot that the miR-378a mimic downregulated GLUT1 protein in PC3 and LNCaP cells, and also downregulated WBP2 in PC3 and, to a lesser extent, LNCaP cells (Fig. 4b, c). Using the miRDB online database [32], we identified putative miR-378a binding sites in the 3’-untranslated regions of the *GLUT1* and *WBP2* mRNAs (Supplementary Table 5), and confirmed that the miR-378a mimic downregulated *GLUT1* and *WBP2* mRNAs (Supplementary Fig. 8f, g). To validate these binding sites, we performed miR-mRNA capture assays [33] by transfecting PC3 and LNCaP cells with a biotinylated miR-378a mimic or non-targeting control, captured the microRNAs on streptavidin-conjugated beads and performed RT-qPCR for *GLUT1* and *WBP2* mRNAs. We confirmed binding of miR-378a to both *GLUT1* and *WBP2* mRNAs (Fig. 4d, e), validating these transcripts as direct targets of this miR.

### GLUT1-facilitated glycolysis is critical for PCa cell survival

**Fig. 5:**
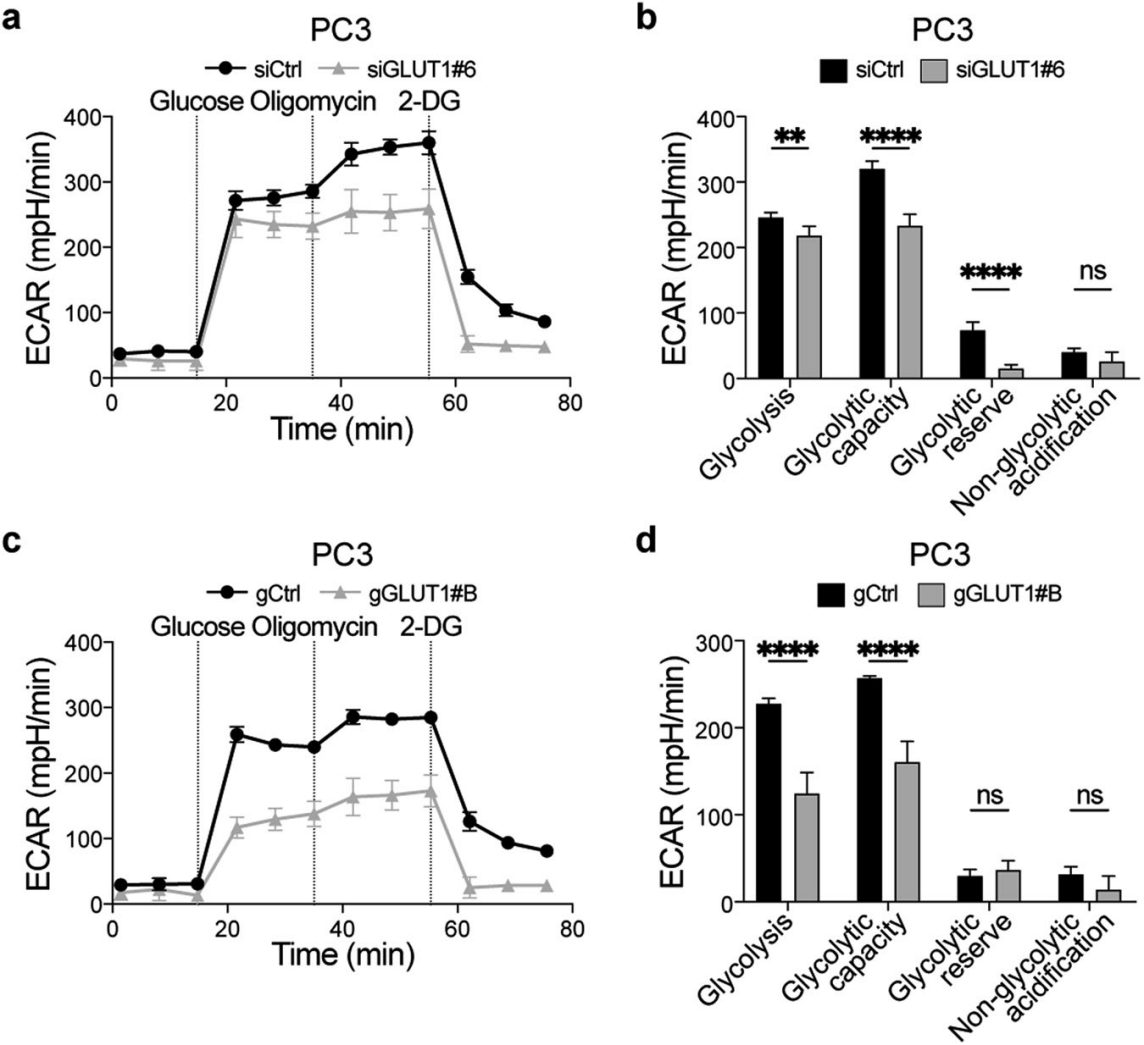
GLUT1 regulates PC3 cell glycolysis and proliferation. **a** Graph showing the extracellular acidification rate (ECAR) in PC3 cells at 72 h after transfection with a *Glut1* targeting siRNA (siGLUT1#6) or a non-targeting control (siCTRL) and challenged with glucose (10 mM), oligomycin (1 µM) and 2-DG (50 mM) as indicated. Values are normalised to total protein content. **b** Quantification of the glyco-stress test parameters using normalised ECAR values relative to baseline from (**a**). Data displayed is from one representative experiment out of three independent biological replicates, with at least five technical replicates each. Results are displayed as mean ± SD; **adjusted *p* ≤ 0.01; ****adjusted *p* ≤ 0.0001, adjusted *p* = ns not significant; unpaired parametric Student’s *t* test using the Holm–Sidak method to correct for multiple comparisons (α = 0.05). **c** Graph showing the extracellular acidification rate (ECAR) in PC3 cells at 72 h after transfection with a *Glut1* targeting gapmeR (gGLUT1#B) or a non-targeting control (gCtrl) and challenged with glucose (10 mM), oligomycin (1 µM) and 2-DG (50 mM) as indicated. Values are normalised to total protein content. **d** Quantification of the glyco-stress test parameters using normalised ECAR values relative to baseline from (**c**). Data displayed is from one representative experiment of three independent biological replicates with at least five technical replicates each. Results are displayed as mean ± SD; ****p ≤ 0.0001, *p* = ns not significant; unpaired parametric Student’s *t* test using the Holm–Sidak method to correct for multiple comparisons (α = 0.05).

**Fig. 6:**
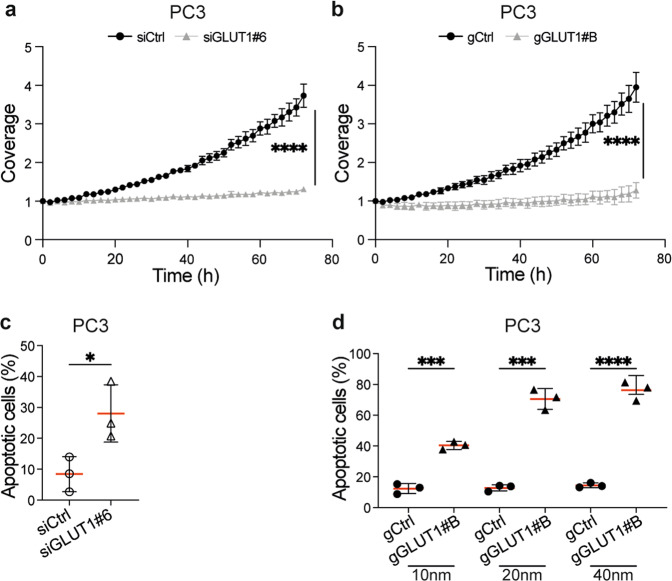
GLUT1 downregulation triggers apoptosis in prostate cancer cells. **a**, **b** Graphs showing PC3 cell growth (coverage in the Incucyte S3 imaging system) 24 h after transfection with siGLUT1#6 or its non-targeting control (siCtrl) (**a**), or with gGLUT1#B or its non-targeting control (gCtrl) (**b**). Data were collected in an Incucyte S3 imaging system for 72 h. Coverage is depicted as the normalised confluency with respect to that observed at day 0. Data are from one representative experiment from three (**a**) or two (**b**) independent biological replicates, each performed with ten technical replicates and are displayed as mean ± SD; ****adjusted *p* ≤ 0.0001 unpaired parametric Student’s *t* test using the Holm–Sidak method to correct for multiple comparisons (α = 0.05). **c**, **d** Graphs showing levels of apoptotic PC3 cells 72 h after transfection with siGLUT1#6 or its non-targeting control (siCtrl) (**c**), or with 10 nM, 20 nM or 40 nM gGLUT1#B or its non-targeting control (gCtrl) (**d**), and analysed by Annexin V/DAPI staining and flow cytometry. Data are from three independent biological replicates; *p ≤ 0.05, ***p ≤ 0.001, ****p ≤ 0.0001; unpaired two-tailed Student’s *t* test.

GLUT1 and WBP2 regulate glucose metabolism [34–36], so to investigate if either of these proteins contribute to the miR-378a metabolic phenotype observed in PCa cells, we transfected PC3 cells with siRNAs targeting WBP2 or GLUT1 (Supplementary Fig. 9a–d). From the four initial siRNA tested, we then selected two siRNAs downregulating WBP2 (siWBP2#7 and siWBP2#8) or GLUT1 (siGLUT1#6 and siGLUT1#8) with different efficiency to test dose-dependency and better address specificity of the approach in functional experiments. WBP2 depletion did not affect glycolysis in PC3 cells (Supplementary Fig. 10a, b). In contrast, GLUT1 depletion suppressed glycolysis in PC3 cells (Fig. 5a, b, Supplementary Fig. 10c, d) and also in LNCaP cells (Supplementary Fig. 10e–h). We confirmed these results in PC3 cells by targeting *GLUT1* mRNA for RNase H-mediated nuclear degradation using gapmeRs (gGLUT1). As a control, gGLUT1#A which did not reduce GLUT1 levels did not affect glycolysis (Supplementary Fig. 11a–d) whereas gGLUT1#B efficiently downregulated GLUT1 and inhibited glycolytic metabolism (Fig. 5c, d). Finally, siRNA and gapmeR-mediated GLUT1 downregulation did not affect the cell cycle (Supplementary Fig. 11e, f), further indicating that miR-378a regulated proliferation and glycolysis via two independent mechanisms. Note however that GLUT1 depletion and reduced glycolysis caused substantial dose-dependent cell death leading to a reduction in the accumulation of viable cells (Fig. 6a–d, Supplementary Fig. 11g, h). These results highlight glucose metabolism as a key process that maintains PCa cell viability, thereby identifying GLUT1 as a potential therapeutic target in highly glycolytic PCa.

## Discussion

Primary cancer lesions often have pre-existing metastatic subclones at diagnosis [9, 10]. We build on that knowledge by examining miR levels in primary PCa from patients presenting local or metastatic disease. We identified a 7-miR signature that is prognostic for DFS in N0 patients independent of age, Gleason score (≤7 vs 8–10), pathological T stage (pT2 vs pT3/pT4), and surgical margin status (negative vs positive). Importantly, this signature also segregates Gleason 7 patients – for whom risk stratification and therapeutic options are uncertain [37] – into higher and lower risk cohorts. Thus, our results confirm that some of the molecular features of patients who will later develop advanced disease can be recognised in early stage primary lesions, facilitating a more assured approach to managing men with intermediate grade disease either with active surveillance or by intervening earlier with radical treatment. Although further validation in new cohorts is needed, we posit that our 7-miR signature could be used alongside histopathological assessment to improve clinical decision-making in patients with localised disease but an uncertain risk of progression.

Functional studies of miRs have identified these molecules as novel actionable targets that could be exploited for therapeutic purposes [38, 39]. Indeed, therapeutic efficacy of targeting miRNAs is being tested in several Phase I clinical trials with encouraging results [40–42]. Here we show that miR-378a expression is significantly decreased in primary PCa compared to normal prostate tissue, and also in high grade (Gleason 8–10) compared to low and intermediate grade (Gleason 6–7) primary tumours. Intriguingly, deregulation of miR-378a and its antisense miR-378a* (or miR-378a-5p) is reported in different cancers, including PCa, resulting in oncogenic or tumour-suppressive effects depending on biological context [30, 43–47]. Accordingly, and in agreement with previous studies [30], we found that increased expression of miR-378a had cytostatic effects resulting in decreased cell proliferation and colony growth, suggesting that low levels of miR-378a may be needed to release the expression of factors required for PCa cell fitness. Interestingly, miR-378a and miR-378a* have been implicated in the regulation of several key metabolic pathways that maintain cellular homeostasis [48–50], and in this regard our proteomic and transcriptomic analyses revealed that miR-378a caused deregulation of several pathways fuelled by glucose catabolism. Interestingly, our mechanistic analysis showed that *Glut1* mRNA is a direct target of miR-378a, indicating that its decrease during transformation releases GLUT1 expression, allowing PCa cells to switch to an increased glycolytic rate. Indeed it is well known that malignant cells reprogram their metabolism to thrive in microenvironments where nutrients are limited [51–54]. Curiously, we found that the inhibition of the glycolytic pathway by miR-378a did not upregulate mitochondrial function and oxidative phosphorylation, a common metabolic adaptation in other cancers [28, 53, 55]. This suggests that unlike other cancers, PCa cells may not switch to mitochondrial respiration to rebalance their energy requirements, supporting our hypothesis that glucose uptake/glucose metabolism could represent unique therapeutic targets in this malignancy.

The data presented herein also advances our understanding of PCa biology and in particular, the energy-related drivers of the metastatic phenotype. In that regard, increased tumour glycolysis is associated with castration-resistant metastatic PCa (mCRPC) and poor prognosis [26, 56], and a rise in glucose consumption marks out neuroendocrine differentiation and metastasis, a known molecular adaptation to androgen-deprivation and AR-targeting therapies [57, 58]. Moreover, previous reports highlighted that GLUT1 expression is higher in poorly differentiated aggressive PCa and is prognostic in patients who progress after radical prostatectomy [59–61]. Furthermore, it has also been suggested that the hypoxic microenvironment may stimulate glucose uptake via GLUT1, allowing the metabolic adaptation to the tumour microenvironment [61, 62]. Notably, we show that GLUT1 inhibition induced pronounced PCa cell death, highlighting the dependency of these cells on glycolysis. These observations are in line with promising results with GLUT1 inhibitors such as WZB117 [35, 63] and BAY-876 [64], and the synergy observed between glucose deprivation and DNA-repair inhibitors in preclinical models [65, 66]. Together, our study provides further support for the implementation of GLUT1 inhibitors in PCa, particularly where miR-378a levels are low and possibly before increased GLUT1 levels are detectable [27].

## Material and methods

### TCGA and MSKCC prostate cancer databases

miRNA expression and the associated clinical data were retrieved from the TCGA Prostate Adenocarcinoma Firehose Legacy (“miRseq_Mature_Preprocess” dataset from http://firebrowse.org/; clinical data from https://www.cbioportal.org) and the Memorial Sloan-Kettering Cancer Center (MSKCC) (https://ftp.ncbi.nlm.nih.gov/geo/series/GSE21nnn/GSE21036/matrix/) databases.

The TCGA data set has been curated as follows: The only metastatic sample (ID #TCGA-V1-A9O5-06A-11R-A41R-13) was removed, leaving 417 samples with available miRNAs expression, N status, disease-free survival (DFS), DFS status and Gleason score (Supplementary Table 1a). The MSKCC dataset [23] (NCBI GEO accession number #GSE21032) included the microRNA expression data (#GSE21036 – normalised log2 miRNA) from 28 non-malignant prostate tissues, 99 primary prostate tumours and 14 metastases. Expression level of hsa-miR-378, sample type (normal, primary, metastasis) and Gleason score were extracted (Supplementary Table 1b).

### Cell lines and culture conditions

Human PCa cell lines 22Rv1 (cat#CRL-2505™), LNCaP (cat#CRL-1740™), PC3 (cat#CRL-1435™) and DU145 (cat#HTB-81™) were from the American Type Culture Collection (ATCC)(Manassas, VA, USA). Human immortalised RWPE-1 cell line (cat#CRL-11609™) was from ATCC and human immortalised benign prostatic hyperplasia BPH-1 cell line (cat#SCC256) from Millipore Sigma (Temecula, CA, USA). Cell lines were STR profiled and tested for mycoplasma. Culture conditions are presented in Supplementary Information.

### RNA transfection

Reverse transfections were performed with 2.5 × 10^5^ cells in 35 mm dishes with 7.5 µl lipofectamine RNAiMAX (Thermo Fisher Scientific, cat#13778150) and Opti-MEM reduced serum medium (Thermo Fisher Scientific, cat# 11058021). Details for miRNAs, siRNAs and gapmers are presented in Supplementary Information.

### Cell growth assay

Transfected cells were seeded at 2.5 × 10^3^ cells/well in a 96-well plates and imaged in an Incucyte S3 Live-Imaging System (Sartorius). Images were captured every 2 h at 10× magnification (Incucyte 10× lens - NA: 0.3) for 3 days, and analysed using the Incucyte S3 software (version 2018c, 2019b and 2020c were used over the course of the experiments).

### Colony formation assay

1 × 10^4^ transfected cells were seeded into 35 mm 6-well plates (Ref # 353046, Corning). After 2 weeks, cells were fixed with 4% paraformaldehyde for 20 min at room temperature (RT), permeabilised with 0.1% Triton X100 in PBS for 20 min (RT), and stained with 0.1% Crystal Violet (Sigma Aldrich, cat# C0775) for 2 h with agitation (RT). Plates were washed with H_2_O, dried (RT) and imaged with GelCount (Oxford Optronix). Area coverage quantification was performed with Image J software (NIH).

### Cell cycle

1 × 10^5^ transfected cells per sample were washed with PBS and fixed in ice cold methanol at −20 °C for at least 24 h. Cells were rehydrated for 10 min with PBS/BSA 0.1%, washed twice with PBS and resuspended for 30 min in PBS containing 1 µg/mL 4′,6-Diamidino-2-phenylindole dihydrochloride (DAPI)(Sigma cat#D9542) and 10 µg/ml RNase A (Thermo Fisher Scientific, cat#EN0531). Subsequently, the cells were washed once in PBS, and resuspended in 500 μl PBS. For each cell population, at least 10,000 cells were analysed in the Novocyte 3000 Flow Cytometer (Acea Bioscience). Single cells were first selected and within those, the proportion of cells in G0/G1, S, and G2/M phases were estimated based on their DNA content using the FlowJo software v10.7.1 (BD).

### Apoptosis assays

1 × 10^5^ transfected cells per sample were washed with PBS and resuspended for 20 min in 100 µl of 1X Annexin-V Binding Buffer (BD Bioscience, cat#556454), 3 µl PE-conjugated Annexin-V antibody (BD Bioscience, cat#560930) and 1 µg/mL DAPI (Sigma cat# D9542). Subsequently, the cells were washed once in PBS, and resuspended in 500 μl PBS. For each cell population, at least 10,000 cells were analysed in the Novocyte 3000 Flow Cytometer (Acea Bioscience). Single cells were first selected and within those, the proportion of Annexin positive cells was determined as the percentage of cells which labelling intensity was above the maximum level of the control. Analysis was performed with FlowJo software v10.7.1 (BD).

### RT-qPCR

Total RNA was extracted from cells using TRIzol (Thermo Fisher Scientific, cat#15596026) according to manufacturer’s instruction, quantified using a NanoDrop Lite Spectrophotometer (Thermo Fisher Scientific) and quality was determined by the OD_260_/OD_280_ ratio. For miR expression analysis, 50 ng total RNA was reverse transcribed to cDNA using TaqMan MicroRNA Reverse Transcription Kit (Thermo Fisher Scientific, cat#4366596) and stored at −20 °C. For gene expression analysis, 500–1000 ng of total RNA was reverse transcribed into cDNA using M-MLV Reverse Transcriptase (Sigma Aldrich, cat#M1302) and stored at −20 °C. Q-PCR analysis was performed using FastStart Universal Probe Master (ROX)(Sigma Aldrich, cat#4913949001). For details see Supplementary Information.

### Protein extraction

Transfected cells were harvested, washed with ice cold PBS and resuspended in RIPA buffer (Sigma Aldrich, cat#R0278) supplemented with cOmplete protease inhibitor cocktail (Sigma Aldrich, cat#11697498001) and PhosSTOP phosphatase inhibitor cocktail (Sigma Aldrich, cat#4906845001). Details for sample processing for isobaric tagged based mass spectrometry and western blots are presented in Supplementary Information.

### Seahorse - extracellular flux analysis

The extracellular acidification rate (ECAR) and the oxygen consumption rate (OCR) were measured independently using a Seahorse XF96 bioanalyzer (Agilent Technologies). Details for sample processing are presented in Supplementary Information.

### Sulforhodamine-B (SRB) staining

SRB staining was used for Seahorse normalisation. On the day of the experiment, the SRB plate was fixed for 1 h with 20% trichloroacetic acid (TCA) (Sigma Aldrich, cat#T6399) at 4 °C. Fixed cells were washed four times with H_2_O, dried at RT and stained for 15 min at RT with 50 μl of 0.4% SRB (Sigma Aldrich, cat#230162) in 1% acetic acid. Stained cells were washed four times with 1% acetic acid, dried at RT and dissolved with 150 μl of 10 mM Tris(hydroxymethyl)-aminomethane. Protein content was measured as optical density at 490 nm using a plate reader.

### Abemaciclib treatment

PC3 and LNCaP cells were treated for 72 h with 200 nM of Abemaciclib (Selleckchem, cat# S5716) or DMSO (vehicle control). Cells were processed for cell cycle analysis and Seahorse extracellular flux as described above.

### Lactate secretion

Transfected cells were seeded in 96-well plates (2 × 10^4^ cells/well) for 72 h. Cells were washed with PBS, then incubated for 1 h at 37 °C with serum-free, phenol-red free RPMI 1640 (Thermo Fisher Scientific, cat#11835030). 10 µl of medium from each replicate was collected, transferred to a new 96-well plate and lactate concentration was quantified using a Lactate Colorimetric/Fluorometric Assay Kit (Biovision, cat#K607) according to the manufacturer’s instructions. Details of sample processing are presented in Supplementary Information.

### MicroRNA pull-down

Cells were transfected with hsa-miR-378a-3p miRCURY LNA miRNA-mimic 3’-biotinylated (Qiagen, cat#YM00471741-BDI-339178) or Negative Control miRCURY LNA miRNA-mimic 3’-biotinylated (Qiagen, cat#YM00479902 – 339178) at 10 nM. After 24 h, the cells were harvested and processed according to the protocol previously described [33] with minor adaptations. Details are presented in Supplementary Information.

### Statistical analysis

Data are expressed as the mean±standard deviation (SD) of at least three independent experiments unless otherwise stated. All statistical tests were performed using GraphPad Prism software (version 9.2.0) and significance displayed as *p ≤ 0.05; **p ≤ 0.01; ***p ≤ 0.001; ****p ≤ 0.0001 (see figures for details). Details for further analyses are presented in Supplementary Information.

## Supplementary information

Supplementary Information

Supplementary Figure 1

Supplementary Figure 2

Supplementary Figure 3

Supplementary Figure 4

Supplementary Figure 5

Supplementary Figure 6

Supplementary Figure 7

Supplementary Figure 8

Supplementary Figure 9

Supplementary Figure 10

Supplementary Figure 11

Supplementary Table 1a

Supplementary Table 1b

Supplementary Table 2

Supplementary Table 3

Supplementary Table 4

Supplementary Table 5

Supplementary Table 6

## References

[1] Bray F, Ferlay J, Soerjomataram I, Siegel RL, Torre LA, Jemal A (2018). Global cancer statistics 2018: GLOBOCAN estimates of incidence and mortality worldwide for 36 cancers in 185 countries. CA Cancer J Clin.

[2] Ziegler A, Koch A, Krockenberger K, Großhennig A (2012). Personalized medicine using DNA biomarkers: a review. Hum Genet.

[3] Xi X, Li T, Huang Y, Sun J, Zhu Y, Yang Y, et al. RNA biomarkers: frontier of precision medicine for cancer. Non Coding RNA. 2017;3. 10.3390/ncrna3010009. 10.3390/ncrna3010009 PMC5832009 29657281

[4] Bartel DP (2018). Metazoan microRNAs. Cell.

[5] Coppola V, De Maria R, Bonci D (2010). MicroRNAs and prostate cancer. Endocr Relat Cancer.

[6] Slack FJ, Chinnaiyan AM (2019). The role of non-coding RNAs in oncology. Cell.

[7] Lan H, Lu H, Wang X, Jin H. MicroRNAs as potential biomarkers in cancer: opportunities and challenges. Biomed Res Int. 2015;2015. 10.1155/2015/125094. 10.1155/2015/125094 PMC4385606 25874201

[8] James ND, Spears MR, Clarke NW, Dearnaley DP, Mason MD, Parker CC, et al. Failure-free survival and radiotherapy in patients with newly diagnosed nonmetastatic prostate cancer. JAMA Oncol. 2016;2. 10.1001/jamaoncol.2015.4350. 10.1001/jamaoncol.2015.4350 PMC4789485 26606329

[9] Gundem G, Van Loo P, Kremeyer B, Alexandrov LB, Tubio JMC, Papaemmanuil E (2015). The evolutionary history of lethal metastatic prostate cancer. Nature.

[10] Cooper CS, Eeles R, Wedge DC, Van Loo P, Gundem G, Alexandrov LB (2015). Analysis of the genetic phylogeny of multifocal prostate cancer identifies multiple independent clonal expansions in neoplastic and morphologically normal prostate tissue. Nat Genet.

[11] Abeshouse A, Ahn J, Akbani R, Ally A, Amin S, Andry CD (2015). The molecular taxonomy of primary prostate cancer. Cell.

[12] Broad Institute of MIT and Harvard. Broad Institute TCGA Genome Data Analysis Center (2016): correlation between miRseq expression and clinical features n.d. 10.7908/C10001K3.

[13] Cerami E, Gao J, Dogrusoz U, Gross BE, Sumer SO, Aksoy BA (2012). The cBio cancer genomics portal: an open platform for exploring multidimensional cancer genomics data. Cancer Disco.

[14] Gao J, Aksoy BA, Dogrusoz U, Dresdner G, Gross B, Sumer SO (2013). Integrative analysis of complex cancer genomics and clinical profiles using the cBioPortal. Sci Signal.

[15] Boorjian SA, Thompson RH, Siddiqui S, Bagniewski S, Bergstralh EJ, Karnes RJ (2007). Long-term outcome after radical prostatectomy for patients with lymph node positive prostate cancer in the prostate specific antigen era. J Urol.

[16] Briganti A, Karnes JR, Da Pozzo LF, Cozzarini C, Gallina A, Suardi N (2009). Two positive nodes represent a significant cut-off value for cancer specific survival in patients with node positive prostate cancer. a new proposal based on a two-institution experience on 703 consecutive N+ patients treated with radical prostatectomy. Eur Urol.

[17] Kane CJ, Eggener SE, Shindel AW, Andriole GL (2017). Variability in outcomes for patients with intermediate-risk prostate cancer (Gleason Score 7, International Society of Urological Pathology Gleason Group 2–3) and implications for risk stratification: a systematic review. Eur Urol Focus.

[18] Coppola V, Musumeci M, Patrizii M, Cannistraci A, Addario A, Maugeri-Saccà M (2013). BTG2 loss and miR-21 upregulation contribute to prostate cell transformation by inducing luminal markers expression and epithelial–mesenchymal transition. Oncogene.

[19] Ribas J, Ni X, Haffner M, Wentzel EA, Salmasi AH, Chowdhury WH (2009). miR-21: an androgen receptor-regulated microRNA that promotes hormone-dependent and hormone-independent prostate cancer growth. Cancer Res.

[20] Mishra S, Deng JJ, Gowda PS, Rao MK, Lin CL, Chen CL (2014). Androgen receptor and microRNA-21 axis downregulates transforming growth factor beta receptor II (TGFBR2) expression in prostate cancer. Oncogene.

[21] Arisan ED, Rencuzogullari O, Freitas IL, Radzali S, Keskin B, Kothari A, et al. Upregulated wnt-11 and mir-21 expression trigger epithelial mesenchymal transition in aggressive prostate cancer cells. Biology. 2020;9. 10.3390/biology9030052. 10.3390/biology9030052 PMC7150874 32182839

[22] Bonci D, Coppola V, Patrizii M, Addario A, Cannistraci A, Francescangeli F (2016). A microRNA code for prostate cancer metastasis. Oncogene.

[23] Taylor BS, Schultz N, Hieronymus H, Gopalan A, Xiao Y, Carver BS (2010). Integrative genomic profiling of human prostate cancer. Cancer Cell.

[24] Bantscheff M, Lemeer S, Savitski MM, Kuster B (2012). Quantitative mass spectrometry in proteomics: Critical review update from 2007 to the present. Anal Bioanal Chem.

[25] Choi SYC, Xue H, Wu R, Fazli L, Lin D, Collins CC (2016). The MCT4 gene: A novel, potential target for therapy of advanced prostate cancer. Clin Cancer Res.

[26] Fox JJ, Gavane SC, Blanc-Autran E, Nehmeh S, Gonen M, Beattie B (2018). Positron emission tomography/computed tomography-based assessments of androgenreceptor expression and glycolytic activity as a prognostic biomarker for metastatic castration-resistant prostate cancer. JAMA Oncol.

[27] Pertega-Gomes N, Felisbino S, Massie CE, Vizcaino JR, Coelho R, Sandi C (2015). A glycolytic phenotype is associated with prostate cancer progression and aggressiveness: a role for monocarboxylate transporters as metabolic targets for therapy. J Pathol.

[28] Shiratori R, Furuichi K, Yamaguchi M, Miyazaki N, Aoki H, Chibana H (2019). Glycolytic suppression dramatically changes the intracellular metabolic profile of multiple cancer cell lines in a mitochondrial metabolism-dependent manner. Sci Rep.

[29] Kawaguchi M, Aoki S, Hirao T, Morita M, Ito K (2016). Autophagy is an important metabolic pathway to determine leukemia cell survival following suppression of the glycolytic pathway. Biochem Biophys Res Commun.

[30] Chen QG, Zhou W, Han T, Du SQ, Li ZH, Zhang Z (2016). MiR-378 suppresses prostate cancer cell growth through downregulation of MAPK1 in vitro and in vivo. Tumor Biol.

[31] Thomson DW, Bracken CP, Goodall GJ (2011). Experimental strategies for microRNA target identification. Nucleic Acids Res.

[32] Chen Y, Wang X (2020). MiRDB: An online database for prediction of functional microRNA targets. Nucleic Acids Res.

[33] Wani S, Cloonan N Profiling direct mRNA-microRNA interactions using synthetic biotinylated microRNA-duplexes. BioRxiv. 2014:0–11. 10.1101/005439.

[34] Ancey PB, Contat C, Meylan E (2018). Glucose transporters in cancer – from tumor cells to the tumor microenvironment. FEBS J.

[35] Liu Y, Cao Y, Zhang W, Bergmeier S, Qian Y, Akbar H (2012). A small-molecule inhibitor of glucose transporter 1 downregulates glycolysis, induces cell-cycle arrest, and inhibits cancer cell growth in vitro and in vivo. Mol Cancer Ther.

[36] Chen S, Zhang Y, Wang H, Zeng YY, Li Z, Li ML, et al. WW domain-binding protein 2 acts as an oncogene by modulating the activity of the glycolytic enzyme ENO1 in glioma article. Cell Death Dis. 2018;9. 10.1038/s41419-018-0376-5. 10.1038/s41419-018-0376-5 PMC5832848 29497031

[37] Preisser F, Cooperberg MR, Crook J, Feng F, Graefen M, Karakiewicz PI, et al. Intermediate-risk prostate cancer: stratification and management. Eur Urol Oncol. 2020:1–11. 10.1016/j.euo.2020.03.002. 10.1016/j.euo.2020.03.002 32303478

[38] Hatziapostolou M, Polytarchou C, Iliopoulos D (2013). MiRNAs link metabolic reprogramming to oncogenesis. Trends Endocrinol Metab.

[39] Ramani R, Megason G, Schallheim J, Karlson C, Vijayakumar V, Vijayakumar S, et al. Integrative analysis of microRNA-mediated gene signatures and pathways modulating white blood cell count in childhood acute lymphoblastic leukemia. Biomark Insights. 2017;12. 10.1177/1177271917702895. 10.1177/1177271917702895 PMC5397277 28469402

[40] Seto AG, Beatty X, Lynch JM, Hermreck M, Tetzlaff M, Duvic M (2018). Cobomarsen, an oligonucleotide inhibitor of miR-155, co-ordinately regulates multiple survival pathways to reduce cellular proliferation and survival in cutaneous T-cell lymphoma. Br J Haematol.

[41] van Zandwijk N, Pavlakis N, Kao SC, Linton A, Boyer MJ, Clarke S (2017). Safety and activity of microRNA-loaded minicells in patients with recurrent malignant pleural mesothelioma: a first-in-man, phase 1, open-label, dose-escalation study. Lancet Oncol.

[42] Beg MS, Brenner AJ, Sachdev J, Borad M, Kang YK, Stoudemire J (2017). Phase I study of MRX34, a liposomal miR-34a mimic, administered twice weekly in patients with advanced solid tumors. Invest N Drugs.

[43] Guo XB, Zhang XC, Chen P, Ma LM, Shen ZQ (2019). MiR‑378a‑3p inhibits cellular proliferation and migration in glioblastoma multiforme by targeting tetraspanin 17. Oncol Rep.

[44] Ding N, Sun X, Wang T, Huang L, Wen J, Zhou Y (2018). MiR-378a-3p exerts tumor suppressive function on the tumorigenesis of esophageal squamous cell carcinoma by targeting Rab10. Int J Mol Med.

[45] Velazquez-Torres G, Shoshan E, Ivan C, Huang L, Fuentes-Mattei E, Paret H, et al. A-to-I miR-378a-3p editing can prevent melanoma progression via regulation of PARVA expression. Nat Commun. 2018;9. 10.1038/s41467-018-02851-7. 10.1038/s41467-018-02851-7 PMC5792646 29386624

[46] Zhang Y, Xu H. Serum exosomal miR-378 upregulation is associated with poor prognosis in non–small-cell lung cancer patients. J Clin Lab Anal. 2020:1–7. 10.1002/jcla.23237. 10.1002/jcla.23237 PMC7307377 32061007

[47] Ell B, Mercatali L, Ibrahim T, Campbell N, Schwarzenbach H, Pantel K (2013). Tumor-induced osteoclast miRNA changes as regulators and biomarkers of osteolytic bone metastasis. Cancer Cell.

[48] Machado IF, Teodoro JS, Palmeira CM, Rolo AP (2020). miR-378a: a new emerging microRNA in metabolism. Cell Mol Life Sci.

[49] Carrer M, Liu N, Grueter CE, Williams AH, Frisard MI, Hulver MW (2012). Control of mitochondrial metabolism and systemic energy homeostasis by microRNAs 378 and 378*. Proc Natl Acad Sci USA.

[50] Liu W, Cao H, Ye C, Chang C, Lu M, Jing Y, et al. Hepatic miR-378 targets p110Î± and controls glucose and lipid homeostasis by modulating hepatic insulin signalling. Nat Commun. 2014;5. 10.1038/ncomms6684. 10.1038/ncomms6684 25471065

[51] McGuirk S, Audet-Delage Y, St-Pierre J (2020). Metabolic fitness and plasticity in cancer progression. Trends Cancer.

[52] Lehúede C, Dupuy F, Rabinovitch R, Jones RG, Siegel PM (2016). Metabolic plasticity as a determinant of tumor growth and metastasis. Cancer Res.

[53] Boudreau A, Purkey HE, Hitz A, Robarge K, Peterson D, Labadie S (2016). Metabolic plasticity underpins innate and acquired resistance to LDHA inhibition. Nat Chem Biol.

[54] Vander Heiden MG, Deberardinis RJ (2017). Understanding the intersections between metabolism and cancer biology CELL-AUTONOMOUS REPROGRAMMING OF CANCER METABOLISM HHS public access. Cell.

[55] de Padua MC, Delodi G, Vucetic M, Durivault J, Vial V, Bayer P (2017). Disrupting glucose-6-phosphate isomerase fully suppresses the “Warburg effect” and activates OXPHOS with minimal impact on tumor growth except in hypoxia. Oncotarget.

[56] Meirelles GSP, Schöder H, Ravizzini GC, Gönen M, Fox JJ, Humm J (2010). Prognostic value of baseline [18F] fluorodeoxyglucose positron emission tomography and99mTc-MDP bone scan in progressing metastatic prostate cancer. Clin Cancer Res.

[57] Rickman DS, Beltran H, Demichelis F, Rubin MA (2017). Biology and evolution of poorly differentiated neuroendocrine tumors. Nat Med.

[58] Aggarwal R, Huang J, Alumkal JJ, Zhang L, Feng FY, Thomas GV (2018). Clinical and genomic characterization of treatment-emergent small-cell neuroendocrine prostate cancer: A multi-institutional prospective study. J Clin Oncol.

[59] Meziou S, Ringuette Goulet C, Hovington H, Lefebvre V, Lavallée É, Bergeron M, et al. GLUT1 expression in high-risk prostate cancer: correlation with 18F-FDG-PET/CT and clinical outcome. Prostate Cancer Prostatic Dis. 2020. 10.1038/s41391-020-0202-x. 10.1038/s41391-020-0202-x 31932660

[60] Stewart GD, Gray K, Pennington CJ, Edwards DR, Riddick ACP, Ross JA (1994). Analysis of hypoxia-associated gene expression in prostate cancer: lysyl oxidase and glucose transporter-1 expression correlate with Gleason score. Oncol Rep.

[61] Jans J, van Dijk JH, van Schelven S, van der Groep P, Willems SH, Jonges TN (2010). Expression and localization of hypoxia proteins in prostate cancer: prognostic implications after radical prostatectomy. Urology.

[62] Williams KJ, Telfer BA, Airley RE, Peters HPW, Sheridan MR, Van der Kogel AJ (2002). A protective role for HIF-1 in response to redox manipulation and glucose deprivation: Implications for tumorigenesis. Oncogene.

[63] Shibuya K, Okada M, Suzuki S, Seino M, Seino S, Takeda H (2015). Targeting the facilitative glucose transporter GLUT1 inhibits the self-renewal and tumor-initiating capacity of cancer stem cells. Oncotarget.

[64] Siebeneicher H, Cleve A, Rehwinkel H, Neuhaus R, Heisler I, Müller T (2016). Identification and optimization of the first highly selective GLUT1 inhibitor BAY-876. ChemMedChem.

[65] Erber J, Steiner JD, Isensee J, Lobbes LA, Toschka A, Beleggia F (2019). Dual inhibition of GLUT1 and the ATR/Chk1 kinase axis displays synergistic cytotoxicity in KRAS-mutant cancer cells. Cancer Res.

[66] Menendez JA, Oliveras-Ferraros C, Cufí S, Corominas-Faja B, Joven J, Martin-Castillo B (2012). Metformin is synthetically lethal with glucose withdrawal in cancer cells. Cell Cycle.

