# Supplementary material for: MiR-378a inhibits glucose metabolism by suppressing GLUT1 in prostate cancer
Source: Oncogene. 2022 Jan 17;41(10):1445–55. doi: 10.1038/s41388-022-02178-0 (PMC8897193; doi:10.1038/s41388-022-02178-0)
Supplement: Supplementary file 1 — Supplementary Information [file 41388_2022_2178_MOESM1_ESM.docx]

**Supplementary Materials and Methods**

*Cell lines*

Cells were maintained at 37°C in a humidified atmosphere with 5% CO_2_ in RPMI 1640 (Thermo Fisher Scientific, cat#31870025; 22Rv1, LNCaP, PC3, BPH-1) or DMEM (ThermoFisher Scientific, cat#41965039; DU145) supplemented with 10% FBS, 100 U/mL penicillin and 100 mg/mL streptomycin (Thermo Fisher Scientific cat#15140122), or in Keratinocyte Serum-free medium (Thermo Fisher Scientific, cat#17005042; RWPE-1) supplemented with 25 mg Bovine Pituitary Extract (BPE), 2.5 µg human recombinant EGF, 100 U/mL penicillin and 100 mg/mL streptomycin.

*RT-qPCR*

The following assays were from Thermo Fisher Scientific: hsa-miR-1-3p (TaqMan assay ID#002222), hsa-miR-21-5p (TaqMan assay ID#000397), hsa-miR-133a-3p (TaqMan assay ID#002246), hsa-miR-217 (TaqMan assay ID#002337), hsa-miR-133b (TaqMan assay ID#002247), hsa-miR-378a-3p (TaqMan assay ID#001314), WBP2 (ID# Hs00600857_m1), SLC2A1/GLUT1 (ID# Hs00892681_m1), GAPDH (ID#4352934E), GPI (ID#Hs00976715), LDHA (ID#Hs01378790), TPI1 (ID#Hs03806547), PGK1 (ID#Hs00943178), ACTB (TaqMan assay ID# Hs01060665_g1) and RNU6B (TaqMan assay ID#001093). Expression of each gene was determined using the 2^−ΔΔCt^ method, and are presented as the fold change relative to ACTB or RNU6B as indicated.

*miRNA mimics, siRNAs and GapmeRrs*

The hsa-miR-378a-3p miRVana miRNA-mimic (cat#MC11360) and negative control #1 (cat#4464058) were from Thermo Fisher Scientific and were used at 10nM; miRIDIAN hsa-miR-378a-3p mimic (cat#C-300686-07) and Negative Control (cat#CN-001000-01) were from Horizon Discovery and were used at 25nM; ON-TARGET plus non-targeting control pool (cat# D-001810-10-05), human SLC2A1/GLUT1 siRNA set of 4 (cat#LQ-007509-02-0005) and human WBP2 siRNA set of 4 (cat# LQ-017572-00-0005) were from Horizon Discovery and were used at 50nM. Antisense LNA negative control A (gCtrl) (cat#LG00000002), LNA SLC2A1/GLUT1 #A (gGLUT1#A) and #B (gGLUT1#B) were from Qiagen and were used at 10nM unless stated otherwise.

*Protein extraction and Western blots*

Cell were lysed in RIPA buffer (Sigma Aldrich, cat#R0278) on ice for 30 min, centrifuged for 30 min at 16000 x g (4 °C) and supernatants were transferred to fresh tubes for protein quantification using the Pierce 660nm protein assay (Thermo Fisher Scientific, cat#2260). Protein denaturation (95 °C for 10 min) was performed with NuPAGE® LDS Sample Buffer (Thermo Fisher Scientific, cat#NP0007) supplemented with NuPAGE® Sample Reducing Agent (Thermo Fisher Scientific, cat#NP0004). Please note that, when samples were interrogated for GLUT1, the same sample processing was followed with the exception of denaturation at 95˚C, according to the manufacturer instructions[1]. SDS-PAGE and transfer were performed on NuPAGE™ 4-12% Bis-Tris Protein Gels (Thermo Fisher Scientific, cat#NP0321BOX) and Trans-Blot® Turbo™ mini (Bio-Rad, cat#1704158) or midi (Bio-Rad, cat#1704159) nitrocellulose membranes. Nonspecific binding sites were blocked with 5% non-fat dried milk or 5% BSA (Sigma Aldrich, cat#A2153) in TBS-0.1% Tween-20 (TBS-T) for 1h (RT with agitation). Primary antibodies were diluted as described below and incubated overnight at 4°C with agitation. HRP-conjugated secondary antibodies were diluted 1:15000 in 5% non-fat dried milk in TBS-T and incubated for 1h at RT in agitation. Immunolabeling was detected with ECL™ Blotting Reagents (Sigma Aldrich, cat#GERPN2109).

The primary antibodies were: anti-Glut1 (Abcam, cat#ab652, dilution 1/1000 in 5% milk/TBS-T), anti-Glut1 (EPR3915) (Abcam, cat#ab115730, dilution 1/10000 in 5% milk/TBS-T), anti-Wbp2 (Santa Cruz Biotechnology, cat#sc-514247, dilution 1/500 in 5% milk/TBS-T), anti-β-Actin (C4) (Santa Cruz Biotechnology, cat#sc-47778, dilution 1/1000 in 5% milk/TBS-T), anti-β-Actin (D6A8) (Cell Signaling Technology, cat#8457S, dilution 1/4000 in 5% BSA/TBS-T), anti-Ccnd1 (92G2) (Cell Signaling Technology, cat#2978, dilution 1/1000 in 5% BSA/TBS-T) and anti-p27 Kip1 (D69C12) XP (Cell Signaling technology, cat#3686, dilution 1/1000 in 5% BSA/TBS-T). The secondary antibodies were: goat anti-mouse IgG antibody (peroxidase)(Jackson ImmunoResearch, cat#115-035-17) and goat anti-rabbit IgG antibody (peroxidase)(Jackson ImmunoResearch, cat#111-035-144). Composite images correspond to a single membrane that was sequentially blotted for the different proteins of interest based on their molecular weight and signal intensity, finalising by blotting for the loading control.

*Seahorse - extracellular flux analysis*

2x10^4^ PC3 or 4x10^4^ LNCaP transfected cells/well were seeded in complete RPMI 1640 medium into 96-well XF96 cell culture microplates (Agilent Technologies, cat# 102601-100) for the assay and, in parallel, into a standard 96-well plate for protein content quantification by SRB and Seahorse normalisation. At 72 hours after transfection, cells in the XF96 plate were incubated with 180 µl of unbuffered Seahorse XF base medium (Agilent Technologies, cat# 102353-100) and incubated at 37°C without CO_2_ for 1h, before the Glycolysis Stress Test and the Mito Stress Test were performed. ECAR was measured under basal conditions followed by sequential addition of 10mM glucose (Sigma Aldrich, cat#G8644), 1 µM oligomycin (Sigma Aldrich, cat#O4876) and 50mM of 2-deoxy-D-glucose (2-DG; Sigma Aldrich, cat#D6134). OCR was measured under basal conditions followed by sequential addition of 1 µM oligomycin, 2µM carbonyl cyanide 4-(trifluoromethoxy)phenylhydrazone (FCCP; Sigma Aldrich, cat#C2920) and 0.5µM Rotenone/Antimycin-A (Rot/AA) (Sigma Aldrich, cat#R8875 and cat#A8674 respectively). Plate normalisation was performed by SRB staining or direct cell lysis (20µl RIPA buffer) on the XF96 plate for protein quantification by Pierce 660nm protein assay.

*Lactate secretion*

Samples were adjusted to 50µl with lactate assay buffer, incubated for 30 min at RT protected from light with 50µl/sample of reaction mix containing lactate assay buffer, lactate assay mix and probe. Fluorescence at 590nm was detected using a SpectraMax M5 plate reader (Molecular devices). Lactate concentration was calculated from a standard curve according to manufacturer’s instructions. Normalisation was performed by SRB protein quantification of the original plate.

*MicroRNA pull-down*

Cells were harvested with gentle scraping, washed in PBS and lysed (10mM KCl, 1.5mM MgCl2, 10mM Tris HCl buffer pH7.5, 5mM DTT, 0.5% IgePal CA-630, 60U/ml SUPERaseIn RNase inhibitor, Complete mini Protease inhibitor 1X) on ice for 30 minutes. For target mRNA capture, the supernatant was retrieved after centrifugation at 16000xg at 4°C and incubated with Dynabeads®MyOne Streptavidin C1 beads (Thermo Fisher Scientific, cat#650-01) for 2h at RT on a rotating mixer. After washing, the RNA:beads mixture was resuspended in 100µL of RNase/DNase free water and RNA extraction was performed using the RNeasy Mini kit (Qiagen, cat#74104) according to manufacturers’ instructions. Purified RNA was analysed for target enrichment by qPCR.

*RNA-Sequencing*

PolyA enriched paired-end libraries of 100bp were sequenced on a HiSeq 2500 (Illumina) in High Throughput mode using TruSeq SBS V3 chemistry (Illumina). After initial quality check with FastQC, adapters from reads were removed using TrimGalore (v0.6.5)[2]. The resultant reads were aligned to GRCh38 using STAR aligner (v2.5.1b)[3]. Gene counts were estimated using featureCounts from the Rsubread package (v2.0.1)[4] and differential expression (DE) between different biological conditions was performed by DESeq2 (v1.26)[5]. The p-values were adjusted using the Benjamini–Hochberg approach for controlling false discovery rate (FDR). Genes with adjusted p-value (Padj) or FDR ≤0.05 were considered to be differentially expressed. RNAseq data described in the publication are available upon request.

*Mass spectrometry*

For protein preparation, transfected cells were lysed using RIPA buffer (Sigma Aldrich, cat#R0278) supplemented with 10μl/ml Benzonase Nuclease (Sigma Aldrich, cat# E1014) and PhosSTOP phosphatase inhibitor cocktail (Sigma Aldrich, cat#4906845001), without protease inhibitors. Samples were incubated for 5 minutes on ice then centrifuged at 16,000 x g for 15 minutes at 4°C. Cleared supernatants were collected and the volume adjusted to give a protein concentration of 1μg/μl.

Dried peptides were dissolved in 200 μL ammonium formate (10 mM, pH 9.5) for high pH RP-Chromatography on a C18 Column (XBridge peptide BEH, 130Å, 3.5 µm 2.1 X 150 mm, Waters) with guard column (XBridge, C18, 3.5 µm, 2.1X10mm, Waters) attached to an Ultimate 3000 HPLC (Thermo-Scientific). Buffer A was 10 mM ammonium formate in milliQ water; buffer B: 10 mM ammonium formate, 90% acetonitrile, both adjusted to pH 9.5 with ammonia. A flow rate of 0.2 ml/min was used and the columns were equilibrated in 2% buffer B for 20 min prior to sample loading (180 µl). The column was washed on 2% buffer B for 1 min and peptides were eluted with a gradient from 2% to 8% buffer B (6 min), 8% to 24% buffer B (25 min), 24% to 25% buffer B (5 min) and finally 25% to 45% buffer B (25 min). The column was washed for 16 min in 100% buffer B and re-equilibrated to 2% buffer B for 20 min. Fractions (200µL, 80 in total) were collected every 1 min using a WPS-3000FC auto-sampler (Thermo-Scientific). The total number of fractions concatenated was set to 12.

Fractions were dried and the peptides were re-suspended in 50 µl 1% formic acid for LC-MS analysis on a Q-Exactive-HF (Thermo Scientific) mass spectrometer coupled with a Dionex Ultimate 3000 RS (Thermo Scientific). Buffer A was 0.1% formic acid in Milli-Q water (v/v); buffer B was 80% acetonitrile in 0.1% formic acid/Milli-Q water (v/v). 5µL aliquots were loaded at 10 μL/min onto a trap column (100 μm × 2 cm, PepMap nanoViper C18 column, 5 μm, 100 Å, Thermo Scientific) equilibrated in 0.1% formic acid. The trap column was washed for 5 min at 10 μL/min with 0.1% formic acid and then switched in-line with a Thermo Scientific, resolving C18 column (75 μm × 50 cm, PepMap RSLC C18 column, 2 μm, 100 Å). The column was maintained at 50°C and peptides were eluted at a constant flow of 300 nl/min with a linear gradient from 2% buffer B to 5% buffer B (5 min), 5% buffer B to 35% buffer B (115 min) and then 35% to 98% buffer B (2 min). The column was washed with 98% buffer B for 15 min and re-equilibrated in 2% buffer B for 21 min. Q-Exactive HF was operated in data dependent positive ionisation mode. The source voltage was 2.4 Kv and the capillary temperature 250°C. A scan cycle comprised MS1 scan (m/z range from 335-1800, with a maximum ion injection time of 50 ms, a resolution of 60,000 and automatic gain control [AGC] value of 3x10^6^) followed by 40 sequential dependant MS2 scans (resolution 7500) of the most intense ions fulfilling predefined selection criteria of AGC 1x10^5^, maximum ion injection time 50 ms, isolation window of 1.4 m/z, fixed first mass of 120 m/z, NCE/Stepped nce27, spectrum data type: centroid, minimum AGC 2.5x10^3^, exclusion of unassigned, singly and >6 charged precursors, peptide match preferred, exclude isotopes on and dynamic exclusion time of 45 s. Mass accuracy was confirmed before initiating sample analysis, and data analysis was performed using Peaks 8.5 based on the parameters in Supplementary Table 6 (https://www.bioinfor.com/wp-content/uploads/2017/08/PEAKS_Studio_85_Manual.pdf; Bioinformatics Solutions Inc, ON, Canada). Mass spectrometry data described in the publication are available upon request.

*Statistical analysis*

Cohorts from the TCGA and MSKCC sequencing datasets for normal tissues, primary tumours and the individual Gleason groups (as indicated in the figures and main manuscript) were analysed for normality using the D’Agostino-Pearson omnibus normality test (significance level of 0.05) and the similarity of their variance was evaluated by the F-Test (significance level of 0.05). All MSKCC cohorts presented a normal distribution with similar variance; thus, comparisons between multiple groups were performed by applying a one-way ANOVA using Tukey correction for multiple comparisons, a multiplicity adjusted p-value for each comparison was determined (significance level of 0.05; 95% confidence interval) and the median with the interquartile range is shown. Regarding the TCGA cohorts, the group corresponding to normal tissue presented a normal distribution whereas the group of the primary tumours did not pass the normality test; thus, downstream comparisons between multiple groups were performed by using the non-parametric Kruskal-Wallis test with Dunn’s correction, a multiplicity adjusted p-value for each comparison was determined (significance level of 0.05) and the median with the interquartile range is shown. Finally, the difference between the median of primary tumours vs normal tissues was done by applying an unpaired, two-tailed Mann-Whitney test (confidence interval=95%) for both the TCGA and MSKCC cohorts; the median difference with the corresponding confidence interval for the difference is reported (95% CI) and the p-values are stated in the figure legends.

Cox proportional-hazards models were used to determine the prognostic ability of the 7-miR signature and individual miRs as predictors of disease-free survival. Multivariable Cox models were adjusted for age, Gleason score (≤7 vs 8-10), pathological T stage (pT2 vs pT3/pT4), N stage (N0 vs N1) and surgical margin status (R0: negative vs R1-R2: positive) predicting disease free survival. All analyses were performed using Stata v13 (StataCorp LP, College Station, TX, USA), patients with N/A in any of the clinical features were not included in the analysis. For details regarding clinical information please refer to Supplementary Table 1a.

**References**

[1] https://www.abcam.com/glucose-transporter-glut1-antibody-epr3915-ab115730.html n.d.

[2] https://www.bioinformatics.babraham.ac.uk/projects/trim_galore/ n.d.

[3] Dobin A, Davis CA, Schlesinger F, Drenkow J, Zaleski C, Jha S, et al. STAR: Ultrafast universal RNA-seq aligner. Bioinformatics 2013;29. https://doi.org/10.1093/bioinformatics/bts635.

[4] Liao Y, Smyth GK, Shi W. The R package Rsubread is easier, faster, cheaper and better for alignment and quantification of RNA sequencing reads. Nucleic Acids Res 2019;47. https://doi.org/10.1093/nar/gkz114.

[5] Love MI, Huber W, Anders S. Moderated estimation of fold change and dispersion for RNA-seq data with DESeq2. Genome Biol 2014;15. https://doi.org/10.1186/s13059-014-0550-8.

**Supplementary figure legends:**

**Supplementary Figure 1: A 7-miR prognostic signature in primary human prostate cancer. a** Unsupervised hierarchical clustering of TCGA N0 and N1 prostate cancer patients using expression (log2 RPM [Reads Per Million]) of the 7 miRs identified from miR RNASeq data. Columns: individual patients, rows: individual miRs. Centering and unit variance scaling are applied to rows, and rows and columns are clustered using correlation distance and average linkage. The dendrogram at the top shows N stage for each patient (N0: green; N1: orange). **b** Bar chart showing the percentage of patients (y-axis) and N0, N1 distribution in Group 1 and Group 2 (x-axis) (see Supplementary Table 1a for details on patient’s IDs; N0: green, N1: orange). ****p≤0.0001; Fisher’s exact test. **c** Kaplan–Meier plot for disease-free survival in Group 1 vs Group 2 molecular subtypes. p=0.0004, HR (95%CI) = 2.6 (1.7 – 4.1); Mantel-Cox test. Median survival Group 1: 71.02 months; median survival Group 2: not reached.

**Supplementary Figure 2: Disease-free survival in PCa patients based on the 7-miR signature. a-f** Kaplan–Meier plots of 5-year disease-free survival of TCGA N0 (localised disease) prostate cancer patients with grouped according to the expression (log2 RPM) levels of miR-21-5p **(a)**, miR-1-3p **(b)**, miR-133b **(c)**, miR-217 **(d)**, miR-139-5p **(e)** and miR-133a-3p **(f)**. The average expression value at baseline of each of the miRs included in the signature was used to assign patients to “low” (below the average) or “high” (above the average) subgroups (see Supplementary Table 1a for details). The number of patients (n) in each group is indicated. *p≤0.05, **p≤0.01, ***p≤0.001, p=NS not significant; Mantel-Cox test.

**Supplementary Figure 3: miR-378a levels are reduced in prostate cancer. a** Graph showing *miR-378a* expression (fold change from comparative Ct of RT-qPCR) normalised to *Rnu6b* in hyperplastic BPH-1 prostate cells, and LNCaP and PC3 prostate cancer cell lines. Data displayed is from three independent biological replicates, each performed in technical triplicates and shown as the mean±SD; **p≤0.01, ***p≤0.001; unpaired Student’s t-test. **b** Graph showing miR-378a expression (log2 RPM) in normal, primary tumours and metastatic samples from the MSKCC dataset. Data is displayed as the median with the interquartile range. Comparisons between multiple groups were performed by applying a one-way ANOVA using Tukey correction for multiple comparisons, and a multiplicity adjusted p-value for each comparison was determined (significance level of 0.05; 95% confidence interval; ****adjusted p≤0.0001). **c** Graph showing the difference of medians between the primary tumours and the normal tissues from the MSKCC dataset. ****p≤0.0001; 95% CI; unpaired two-tailed Mann-Whitney test. **d** Graph showing miR-378a expression (log2 RPM) in primary prostate cancer from the MSKCC dataset according to Gleason score (G5/6: Gleason 5 and 6; G7: Gleason 7; G8/9: Gleason 8 and 9). Data is displayed as the median with the interquartile range. Comparisons between multiple groups were performed by applying a one-way ANOVA using Tukey correction for multiple comparisons, a multiplicity adjusted p-value for each comparison was determined (significance level of 0.05; 95% confidence interval; *adjusted p≤0.05). (**b, d**) Only statistically significant changes are displayed.

**Supplementary Figure 4: miR-378a regulates the metabolism of the LNCaP prostate cancer cell line. a** Enrichment plots from gene set enrichment analysis (GSEA software v4.1.0; Hallmark gene set: h.all.v7.4.symbols.gmt; KEGG gene set: c2.cp.kegg.v7.4.symbols.gmt) of RNAseq from PC3 (left) and LNCaP (right) cells, 72h after transfection with a miR-378a mimic or non-targeting control. Differentially expressed genes were considered as significant for padj≤0.05. The Normalized Enrichment Score (NES), nominal p-value and FDR q-value are indicated on the graphs. A gene set is considered significantly enriched if its NES has an FDR q-value ≤ 0.25. **b** Graph showing the expression changes of genes related to glycolysis (HALLMARK_GLYCOLYSIS) (x axis) in LNCaP (white bar) and PC3 (grey bar) cells transfected with a miR-378a mimic compared to the non-targeting control (log2 fold change from normalised counts of the RNAseq dataset – y axis). The 46 glycolysis-related genes displayed correspond to the genes differentially expressed in both PC3 and LNCaP cells among the 200 genes of the glycolysis hallmark gene set (https://www.gsea-msigdb.org/gsea/msigdb/cards/HALLMARK_GLYCOLYSIS.html). Data presented is from three independent biological replicates and is shown as the log2 ratio of the mean±SE; *p≤0.05, **p≤0.01; ***p≤0.001, ****p≤0.0001; unpaired two-tailed Student’s t-test. **c** Graph showing the extracellular acidification rate (ECAR) in LNCaP cells 72h after transfection with a miR-378a-mimic or non-targeting control (Ctrl) and then challenged with glucose (10mM), oligomycin (1µM) and 2-DG (50mM). Values are normalised to total protein content. **d** Quantification of the glyco-stress test parameters in LNCaP cells from the normalised ECAR values displayed in **(c)**. One representative experiment out of three independent biological replicates with ten technical replicates each is shown as the mean±SD; *adjusted p≤0.05, ****adjusted p≤0.0001; unpaired parametric Student’s t-test using the Holm-Sidak method to correct for multiple comparisons (α=0.05). **e** Quantification of extracellular lactate in LNCaP cells 72h after transfection with a miR-378a-mimic or a non-targeting control (Ctrl). Data displayed is from one biological experiment with four independent technical replicates. Results are shown as the mean±SD; *p≤0.05; unpaired Student’s t-test.

**Supplementary Figure 5: Oxygen consumption rate (OCR) does not increase upon blocking glycolysis in PCa cells. a** Graph showing the oxygen consumption rate (OCR) in PC3 cells 72h after transfection with the miR-378a-mimic or non-targeting control and challenged with oligomycin (1µM), FCCP (2µM) and Rot/AA (0.5µM). Values are normalised to total protein content. **b** Quantification of the mito-stress test parameters in PC3 cells using the normalised OCR values for each time point shown in **(a)**. One representative experiment out of three independent biological replicates with ten technical replicates each is shown as the mean±SD; p=ns not significant; unpaired parametric Student’s t-test using the Holm-Sidak method to correct for multiple comparisons (α=0.05). **c** Graph showing the oxygen consumption rate (OCR) in LNCaP cells 72h after transfection with the miR-378a-mimic or non-targeting control and then challenged with oligomycin (1µM), FCCP (2µM) and Rot/AA (0.5µM). Values are normalised to the total protein content. **d** Quantification of the mito-stress test parameters in LNCaP cells using the normalised OCR values for each time point shown in **(c)**. One representative experiment out of three independent biological replicates with ten technical replicates each is shown as the mean±SD; p=ns not significant; unpaired parametric Student’s t-test using the Holm-Sidak method to correct for multiple comparisons (α=0.05).

**Supplementary Figure 6: miR-378a promotes cell cycle arrest and inhibits proliferation in PCa cells. a** Representative Western Blot for CCND1 and P27 with β-Actin (ACTB) as the loading control in PC3 and LNCaP cells 72h after transfection with the miR-378a-mimic or non-targeting control (Ctrl). **b** Cell cycle analysis of PC3 and LNCaP cells 72h after transfection with the miR-378a-mimic or non-targeting control (Ctrl). Data from each cell cycle phase from at least three independent biological replicates is shown as the mean±SD. **adjusted p≤0.01, ***adjusted p≤0.001; unpaired parametric Student’s t-test using the Holm-Sidak method to correct for multiple comparisons (α=0.05). **c** Graph showing the growth of PC3 cells (coverage in Incucyte S3 imaging system) following transfection with the miR-378a mimic (grey) or non-targeting control (black) for 24h, followed by imaging for a further 72h. Coverage rate is depicted as the normalised confluency with respect to that observed at day 0. Data from one representative experiment out of three independent biological replicates with at least 5 technical replicates each is presented as the mean±SD. ****adjusted p<0.0001; unpaired parametric Student’s t-test using the Holm-Sidak method to correct for multiple comparisons (α=0.05). **d** Colony formation assays in PC3 cells transfected with the miR-378a-mimic (grey) or non-targeting control (black). The area of coverage in cells transfected with the miR-378a mimic relative to the non-targeting control (Ctrl) is shown. Data from three independent biological replicates with three technical replicates each is presented as the mean±SD. ****p≤0.0001; unpaired two-tailed Student’s t-test. **e** Graph showing the LNCaP cell growth (coverage in the Incucyte S3 imaging system) following transfection with the miR-378a-mimic (grey) or a non-targeting control (black) for 24h, followed by imaging for a further 72h. Coverage is depicted as the normalised confluency with respect to that observed at day 0. Data from one representative experiment out of two independent biological replicates with at least 5 technical replicates each is displayed as the mean±SD. ****adjusted p≤0.0001; unpaired parametric Student’s t-test using the Holm-Sidak method to correct for multiple comparisons (α=0.05). **f** Colony formation assays in LNCaP cells transfected with the miR-378a mimic (grey) or non-targeting control (black). The area of coverage in cell transfected with the miR-378a mimic relative to the non-targeting control (Ctrl) is shown. Data from two independent biological replicates with three technical replicates each is presented as mean±SD. ****p≤0.0001; unpaired, two-tailed Student’s t-test.

**Supplementary Figure 7: Abemaciclib-mediated cell cycle arrest does not affect the glycolytic rate of PCa cells. a, b** Growth inhibitory effect of abemaciclib in PC3 **(a)** or LNCaP **(b)** cells. Cell growth was measured by sulforhodamine-B staining at 72h after treatment with abemaciclib and normalised to that of the vehicle treated control. Each data point represents the mean±SD of three replicates from one biological experiment. **c, d** Cell cycle of PC3 **(c)** or LNCaP cells **(d)** treated with 200nM of abemaciclib for 72h. **e, f** Quantification of the glyco-stress test parameters using the normalised ECAR values for each time point in PC3 **(e)** or LNCaP **(f)** cells treated for 72h with vehicle or abemaciclib. Data from one experiment with ten replicates is displayed as the mean±SD. *adjusted p≤0.05, **adjusted p≤0.01, p=ns not significant; unpaired parametric Student’s t-test using the Holm-Sidak method to correct for multiple comparisons (α=0.05).

**Supplementary Figure 8: miR-378a mediates GLUT1 and WBP2 downregulation in PCa cells. a,** **b** Graphs showing WBP2 (**a**) and GLUT1 (**b**) intensity measured by TMT-mass spectrometry in PC3 cells 72h after transfection with the miR-378a-mimic or non-targeting control. Data from three independent biological experiments is shown as the mean±SD. *p≤0.05, ****p≤0.0001; unpaired Student’s t-test. The difference between means ±SD is plotted on the right of each graph. **c** Volcano plot showing significant changes (fold change) in proteins identified by mass-spectrometry in LNCaP cells 72h after transfection with the miR-378a-mimic compared to the non-targeting control. Differentially expressed proteins were filtered using a cut-off of “unique-peptides/number-of-peptides” ratio ≥ 0.25 and a PEAKS significance value ≥5. The orange dot highlights GLUT1. **d,** **e** Graphs showing GLUT1 (**d**) and WBP2 (**e**) intensity measured by TMT-mass spectrometry in LNCaP cells 72h after transfection with a miR-378a-mimic or non-targeting control. Data from three independent biological experiment is shown as the mean±SD. *p≤0.05, p=ns not significant; unpaired Student’s t-test. The difference between means ±SD is plotted on the right of each graph. **f, g** Graphs showing expression of *Glut1* **(f)** or *Wbp2* **(g)** mRNAs normalised to *Actb* (fold change from comparative Ct values, RT-qPCR) in PC3 and LNCaP cells 72h after transfection with the miR-378a-mimic or non-targeting control (Ctrl). Data from four independent biological replicates performed in three technical replicates each is shown as the mean±SD. *p≤0.05, **p≤0.01, ****p≤0.0001; unpaired Student’s t-test.

**Supplementary Figure 9: siRNA mediated downregulation of WBP2 and GLUT1 in PCa cells. a** Graph showing *Wbp2* mRNA expression normalised to *Actb* (fold change from comparative Ct values, RT-qPCR) in PC3 cells 72h after transfection with four *WBP2* siRNAs (siWBP2#5-8) or a non-targeting control (siCtrl). Data from three technical replicates of one biological experiment is shown as the mean±SD. ***p≤0.001; ****p≤0.0001; unpaired Student’s t-test. **b** Western blot for WBP2 and β-Actin (ACTB) as the loading control 72h after PC3 cell transfection with the four WBP2 siRNAs or non-targeting control (siCtrl) from **(a)**. **c** Graph showing *Glut1* mRNA expression normalised to *Actb* (fold change from comparative Ct values, RT-qPCR) in PC3 cells 72h after transfection with four *GLUT1* siRNAs (siGLUT1#6-8,18) or a non-targeting control (siCtrl). Data from three technical replicates of one biological experiment is shown as the mean±SD. ****p≤0.0001 and p=ns not significant; unpaired Student’s t-test. **d** Western blot for GLUT1 and β-Actin (ACTB) as loading control 72h after PC3 cell transfection with the four different on-target GLUT1 siRNAs or a non-targeting control (siCtrl) from **(c)**.

**Supplementary Figure 10: GLUT1 downregulation affects PCa cell glycolysis. a** Graph showing extracellular acidification rate (ECAR) in PC3 cells 72h after transfection with siWBP2#7, siWBP2#8 or a non-targeting control and challenged with glucose (10mM), oligomycin (1µM) and 2-DG (50mM). Values are normalised to the total protein content. **b** Quantification of the glyco-stress test parameters from the normalised ECAR values from **(a)**. Data from one biological experiment with 10 replicates is displayed as the mean±SD; **adjusted p≤0.01; ****adjusted p≤0.0001; unpaired parametric Student’s t-test using the Holm-Sidak method to correct for multiple comparisons (α=0.05). **c** Graph showing extracellular acidification rate (ECAR) in PC3 cells 72h after transfection with a *GLUT1* targeting siRNA (siGLUT1#8) or a non-targeting control (siCtrl) and challenged with glucose (10mM), oligomycin (1µM) and 2-DG (50mM). Values are normalised to total protein content. **d** Quantification of the glyco-stress test parameters using normalised ECAR values relative to baseline from **(c)**. Data from one biological experiment with five replicates is displayed as the mean±SD; *adjusted p≤0.05, ****adjusted p≤0.0001; unpaired parametric Student’s t-test using the Holm-Sidak method to correct for multiple comparisons (α=0.05). **e** Graph showing *Glut1* mRNA expression normalised to *Actb* (fold change from comparative Ct values, RT-qPCR) in LNCaP cells 72h after transfection with four *Glut1* siRNAs (siGLUT1#6-8,18) or a non-targeting control (siCtrl). Data from three technical replicates of one biological experiment is shown as the mean±SD. **p≤0.01, ****p≤0.0001; unpaired Student’s t-test. **f** Western blot for GLUT1 and β-Actin (ACTB) as the loading control 72h after LNCaP cell transfection with the four GLUT1 siRNAs or a non-targeting control (siCtrl) from **(e)**. **g** Graph showing extracellular acidification rate (ECAR) in LNCaP cells 72h after transfection with a *GLUT1* targeting siRNA (siGLUT1#6) or a non-targeting control (siCtrl) and challenged with glucose (10mM), oligomycin (1µM) and 2-DG (50mM). Values are normalised to total protein content. **h** Quantification of the glyco-stress test parameters using normalised ECAR values relative to baseline from **(g)**. Data from one biological experiment with ten replicates is displayed as the mean±SD; ****adjusted p≤0.0001, adjusted p=ns not significant; unpaired parametric Student’s t-test using the Holm-Sidak method to correct for multiple comparisons (α=0.05).

**Supplementary Figure 11: Effect of GLUT1 downregulation in PCa cells. a** Graph showing *Glut1* mRNA expression normalised to *Actb* (fold change from comparative Ct values, RT-qPCR) in PC3 cells 72h after transfection with gGLUT1#A (#A), gGLUT1#B (#B) or a non-targeting control (gCtrl). Data from one biological experiment with two technical replicates is represented as the mean±SD. *p≤0.05, ***p≤0.001; unpaired Student’s t-test. **b** Western blot for GLUT1 and β-Actin (ACTB) as loading control in PC3 cells 72h after transfection with gGLUT1#A, gGLUT1#B or a non-targeting control (gCtrl). **c** Graph showing extracellular acidification rate (ECAR) in PC3 cells 72h after transfection with a *GLUT1* targeting gapmeR (gGLUT1#A) or non-targeting control (gCtrl) and challenged with glucose (10mM), oligomycin (1µM) and 2-DG (50mM). Values are normalised to total protein content. **d** Quantification of the glyco-stress test parameters using normalised ECAR values relative to baseline from **(c)**. Data from one biological experiment with five replicates is displayed as the mean±SD; adjusted p=ns not significant; unpaired parametric Student’s t-test using the Holm-Sidak method to correct for multiple comparisons (α=0.05). **e** Cell cycle analysis of PC3 cells 72h after transfection with siGLUT1#6 or a non-targeting control (siCtrl). Data from three biological experiments is shown as the mean±SD; p=ns not significant; unpaired parametric Student’s t-test using the Holm-Sidak method to correct for multiple comparisons (α=0.05). **f** Cell cycle analysis of PC3 cells 72h after transfection with gGLUT1#B or a non-targeting control (gCTRL). Data from two biological experiments is shown as the mean±SD; p=ns not significant; unpaired parametric Student’s t-test using the Holm-Sidak method to correct for multiple comparisons (α=0.05). **g, h** Graphs showing the growth (coverage in Incucyte S3 imaging system) of PC3 cells 24h after transfection with a *GLUT1* targeting siRNA (siGLUT1#8; grey) and non-targeting control (siCtrl; black) **(g),** or a *GLUT1* targeting gapmeR (gGLUT1#A; grey) and non-targeting control (gCTRL; black) **(h)** and imaged for 72h. Growth is depicted as the normalised confluency with respect to day 0. Data from 10 technical replicates is presented as the mean±SD. ****adjusted p≤0.0001 **(g)**, p=ns not significant **(h)**; unpaired parametric Student’s t-test using the Holm-Sidak method to correct for multiple comparisons (α=0.05).

**Supplementary table legends:**

**Supplementary Table 1a: miRNA expression and associated clinical data from the TCGA prostate adenocarcinoma database.**

**Supplementary Table 1b:** **miRNA expression and associated clinical data from the MSKCC prostate cancer database.**

**Supplementary Table 2: Top 10 miRNAs differentially expressed in N1 vs N0 PCa patients.** NA: undetected cases; AUC: Area Under the Curve.

**Supplementary Table 3:** **Expression levels of the indicated miRs in non-malignant and cancerous prostate epithelial cell lines.** The mean±SD of RTqPCR raw Ct values for the indicated miRs and RNU6B (normalisation control) are displayed. ND: not detected.

**Supplementary Table 4: Expression levels of miR-378-3p in non-malignant and cancerous prostate epithelial cell lines.** The mean±SD of RTqPCR raw Ct values for miR-378a-3p and RNU6B (normalisation control) are displayed.

**Supplementary Table 5: Predicted miR-378a binding sites in GLUT1 and WBP2 3'UTRs.**

**Supplementary Table 6: Parameters used for the analysis of the TMT mass spectrometry datasets with PEAKS 8.5.**
