# Supplementary figures and images for: MiR-378a inhibits glucose metabolism by suppressing GLUT1 in prostate cancer
Source: Oncogene. 2022 Jan 17;41(10):1445–55. doi: 10.1038/s41388-022-02178-0 (PMC8897193; doi:10.1038/s41388-022-02178-0)

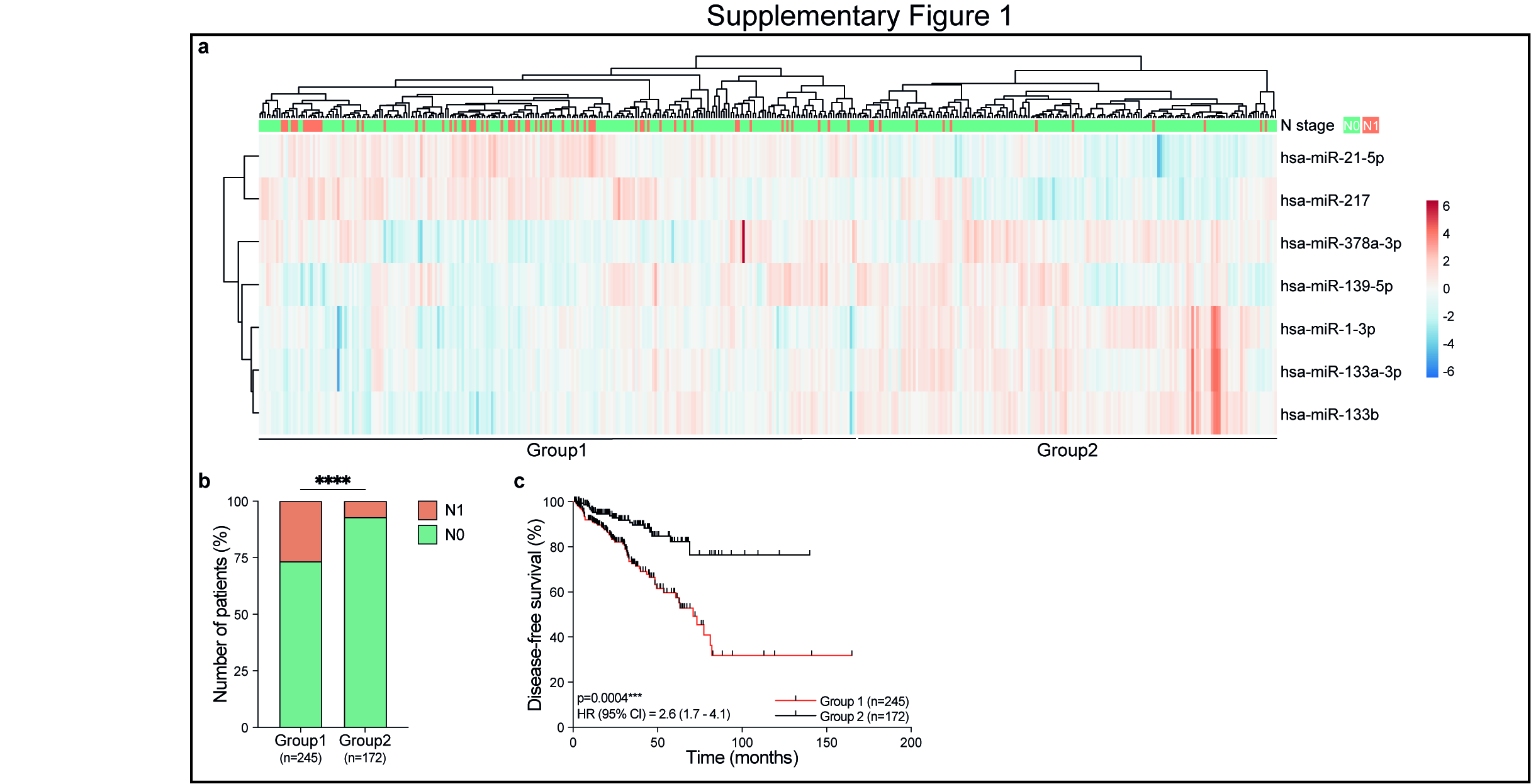

Supplement: Supplementary file 2 — Supplementary Figure 1 [file 41388_2022_2178_MOESM2_ESM.tif]

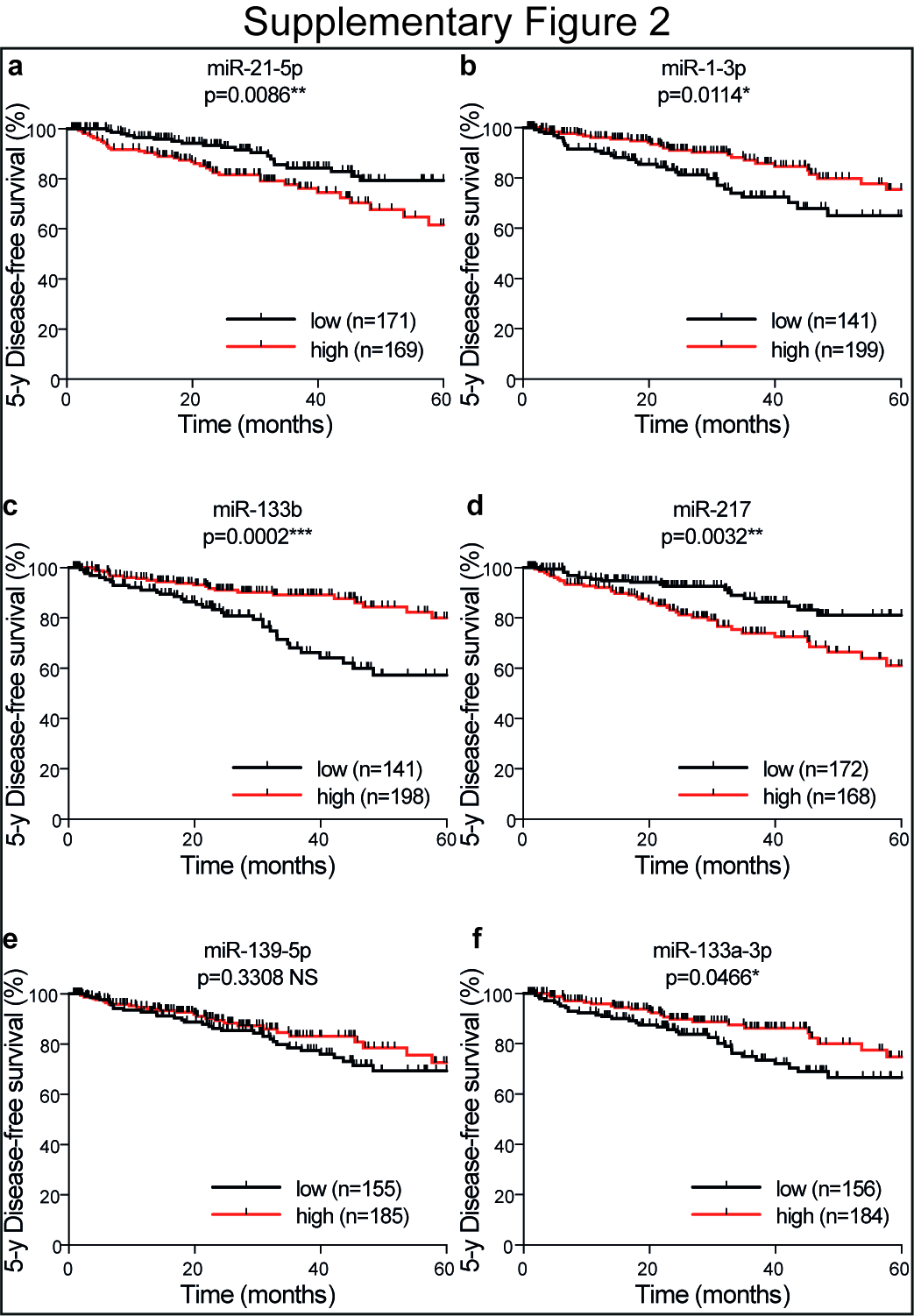

Supplement: Supplementary file 3 — Supplementary Figure 2 [file 41388_2022_2178_MOESM3_ESM.tif]

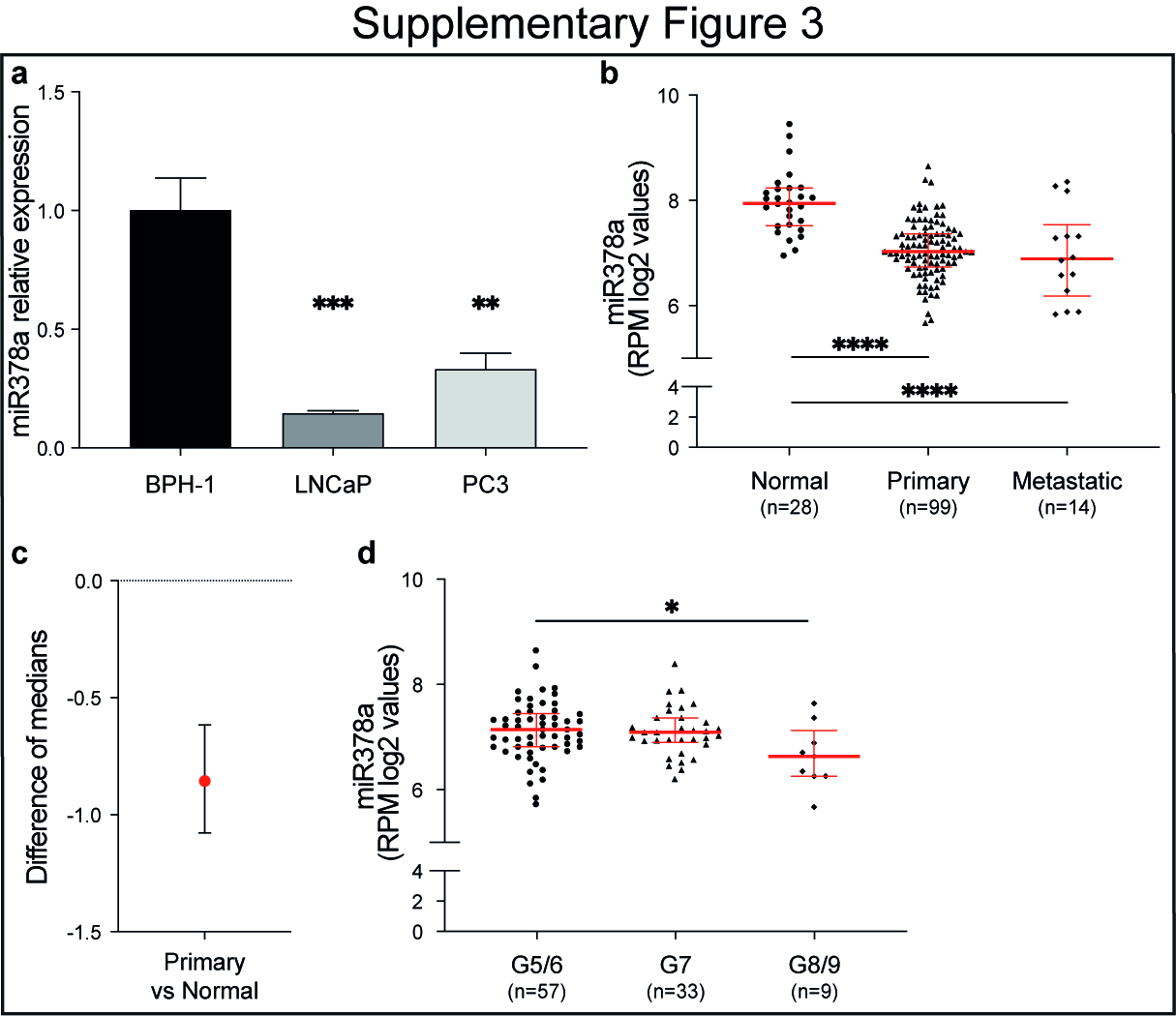

Supplement: Supplementary file 4 — Supplementary Figure 3 [file 41388_2022_2178_MOESM4_ESM.tif]

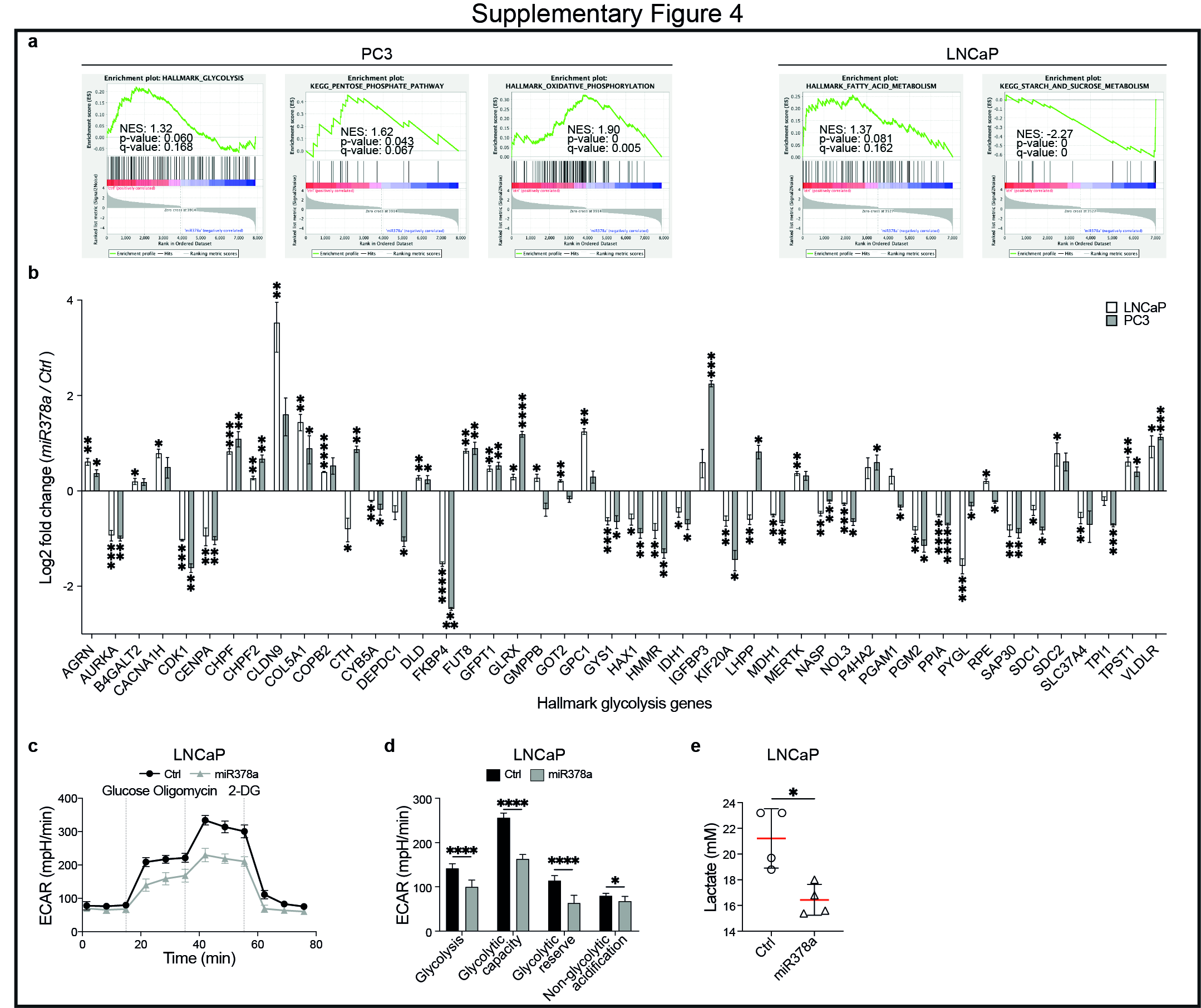

Supplement: Supplementary file 5 — Supplementary Figure 4 [file 41388_2022_2178_MOESM5_ESM.tif]

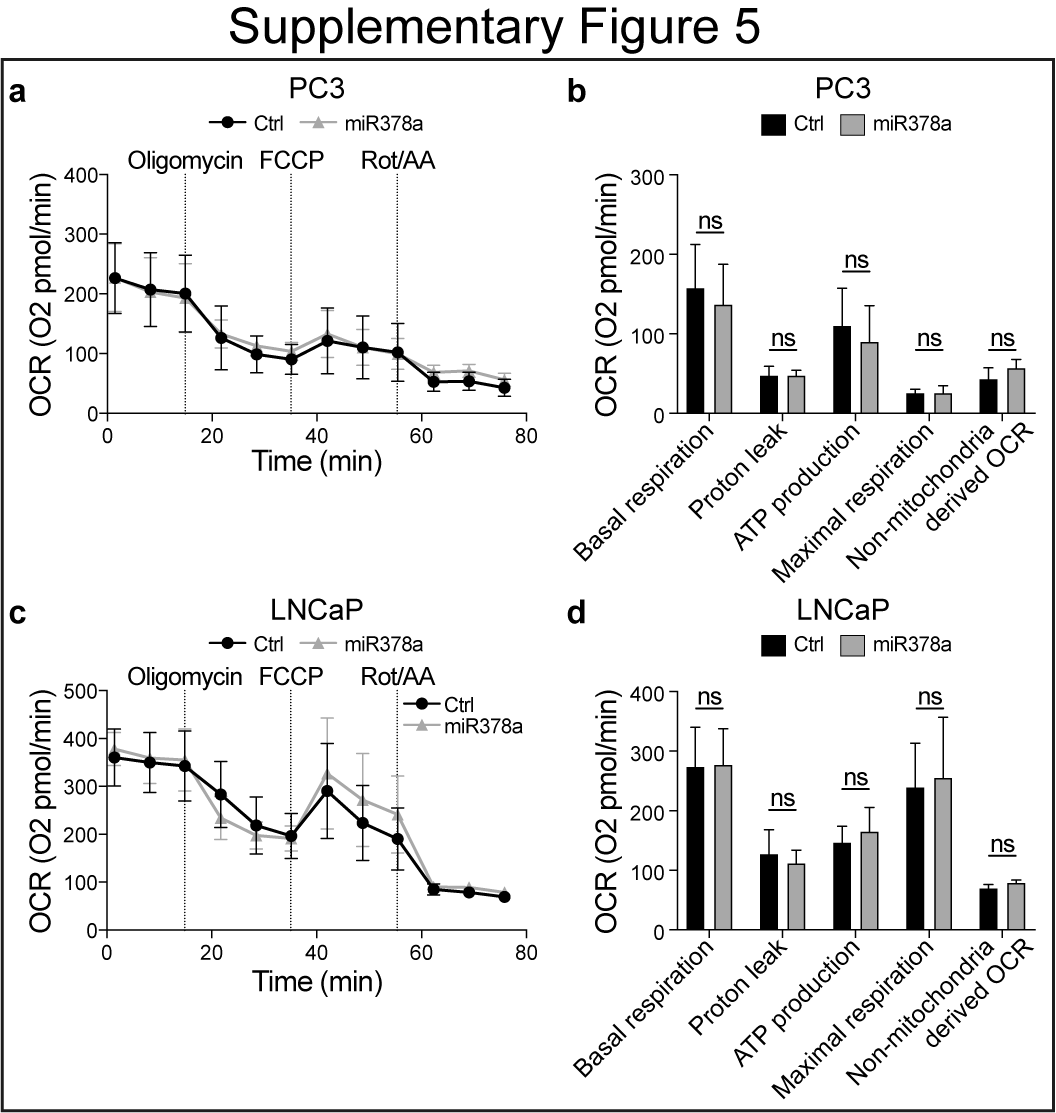

Supplement: Supplementary file 6 — Supplementary Figure 5 [file 41388_2022_2178_MOESM6_ESM.tif]

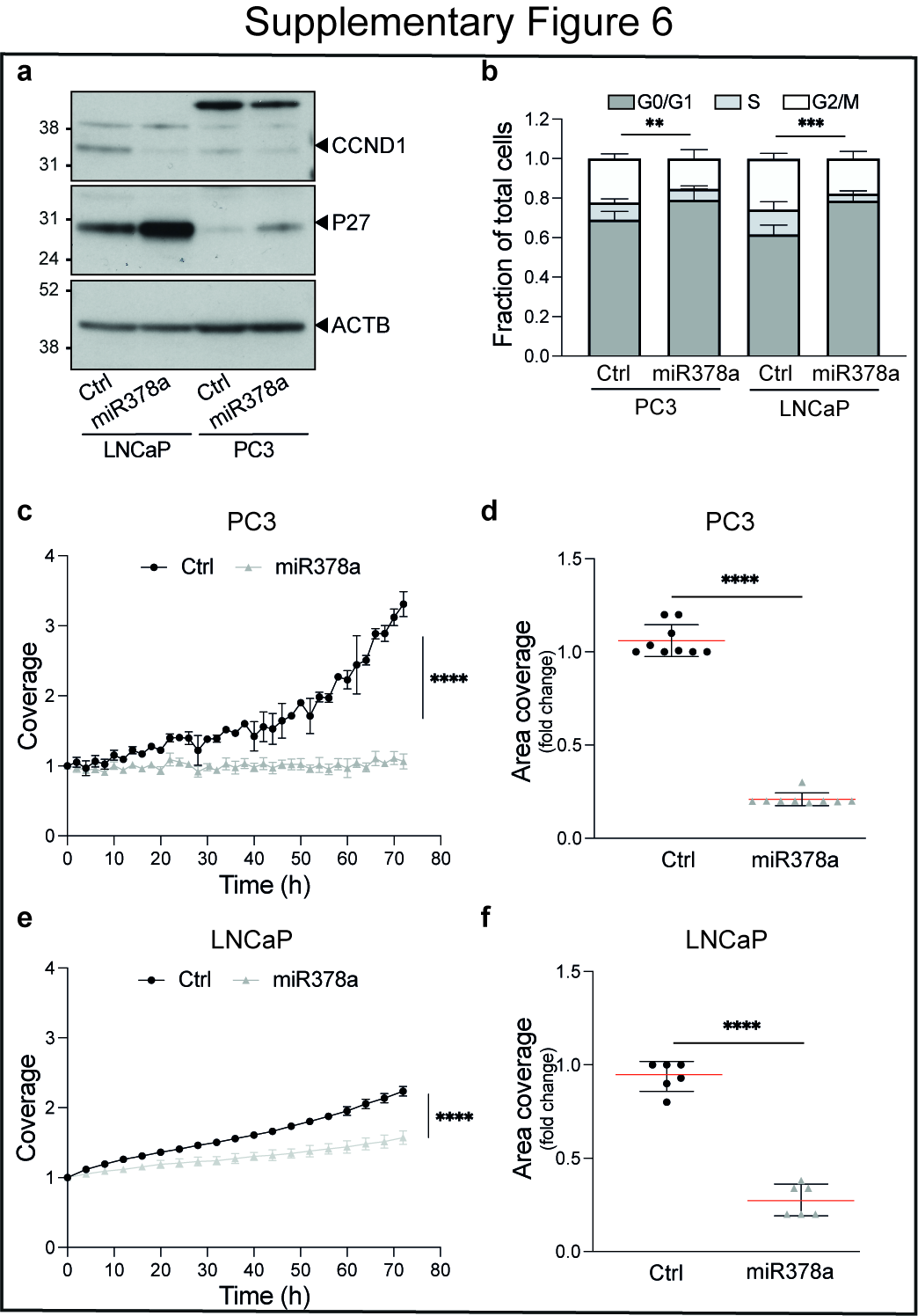

Supplement: Supplementary file 7 — Supplementary Figure 6 [file 41388_2022_2178_MOESM7_ESM.tif]

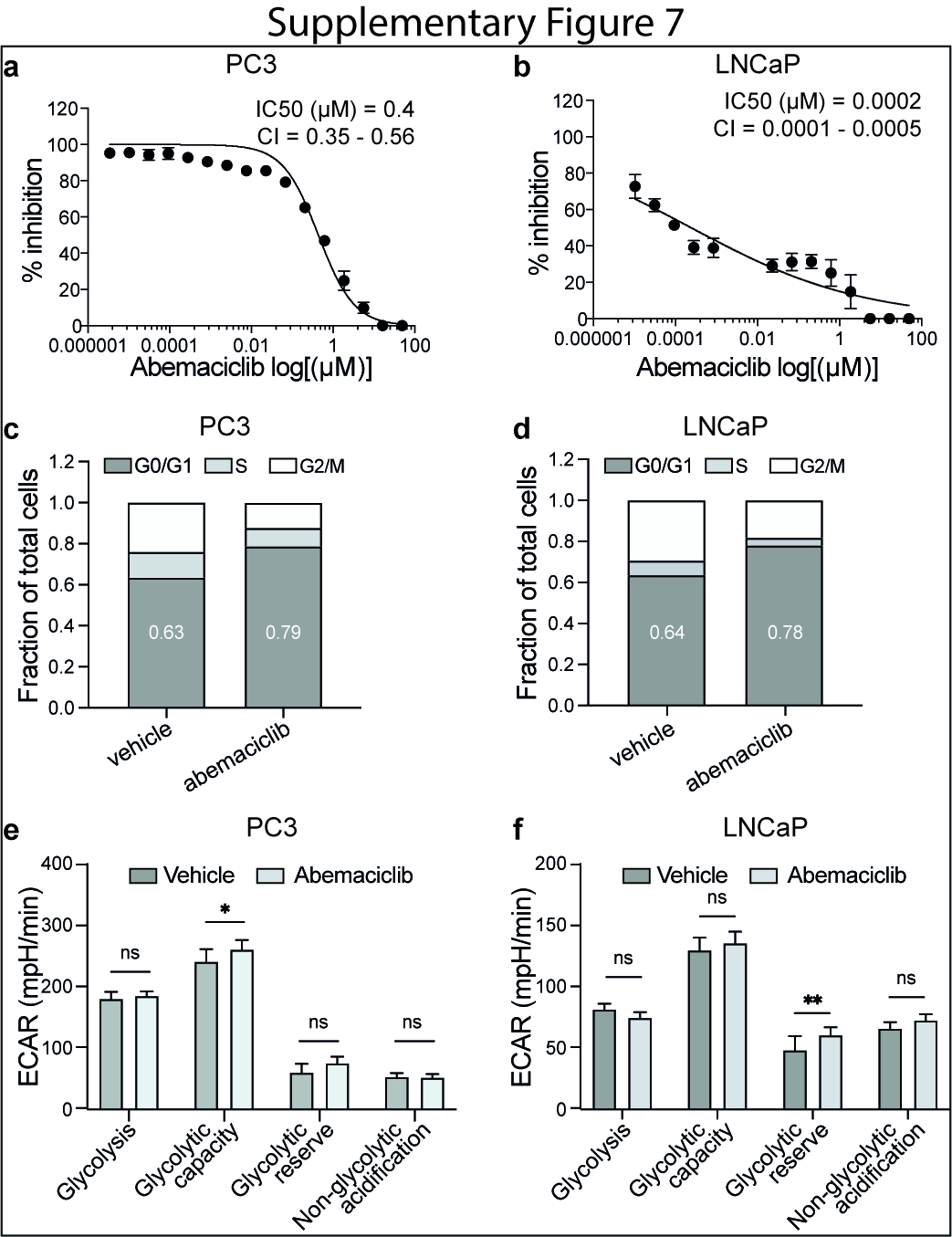

Supplement: Supplementary file 8 — Supplementary Figure 7 [file 41388_2022_2178_MOESM8_ESM.tif]

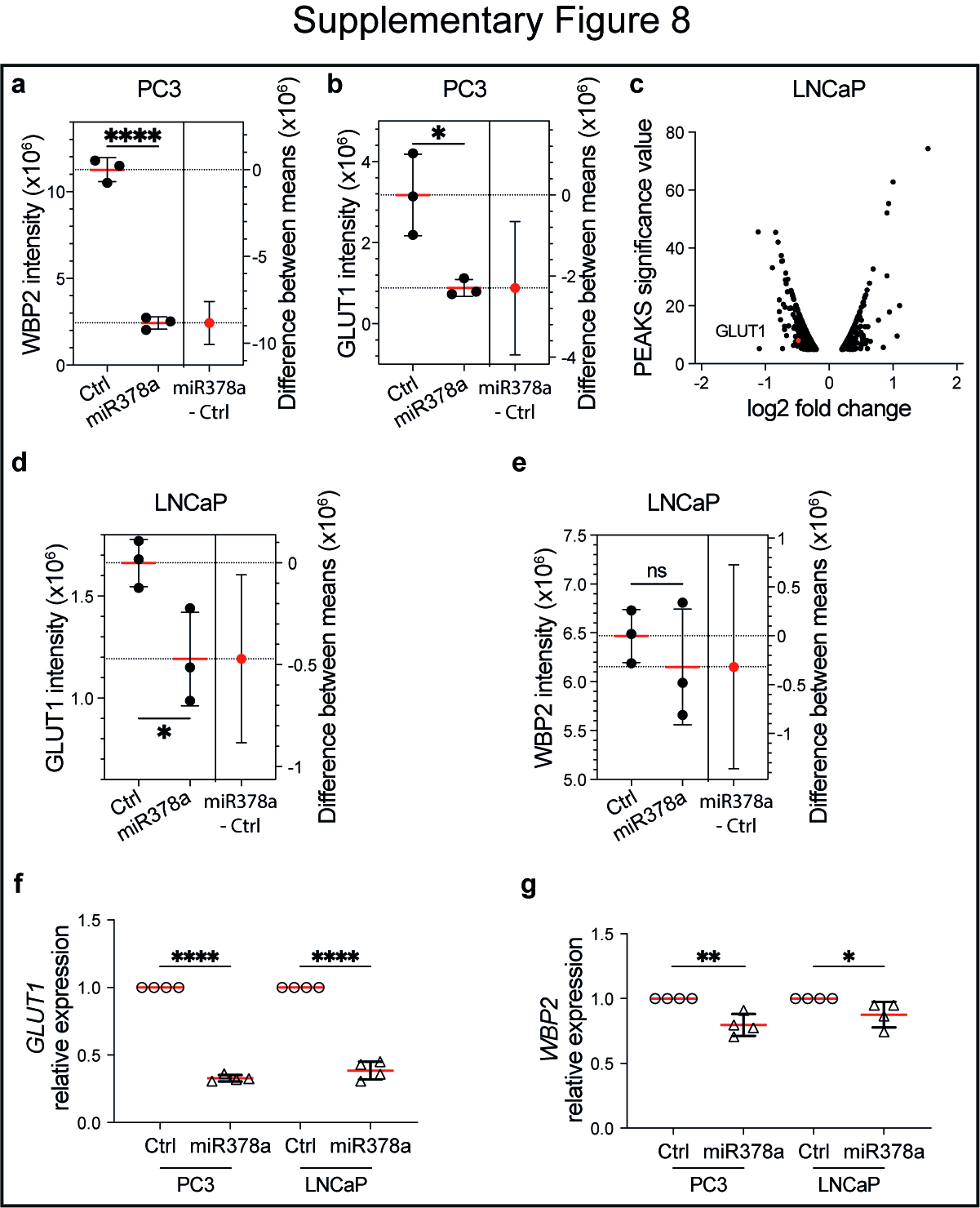

Supplement: Supplementary file 9 — Supplementary Figure 8 [file 41388_2022_2178_MOESM9_ESM.tif]

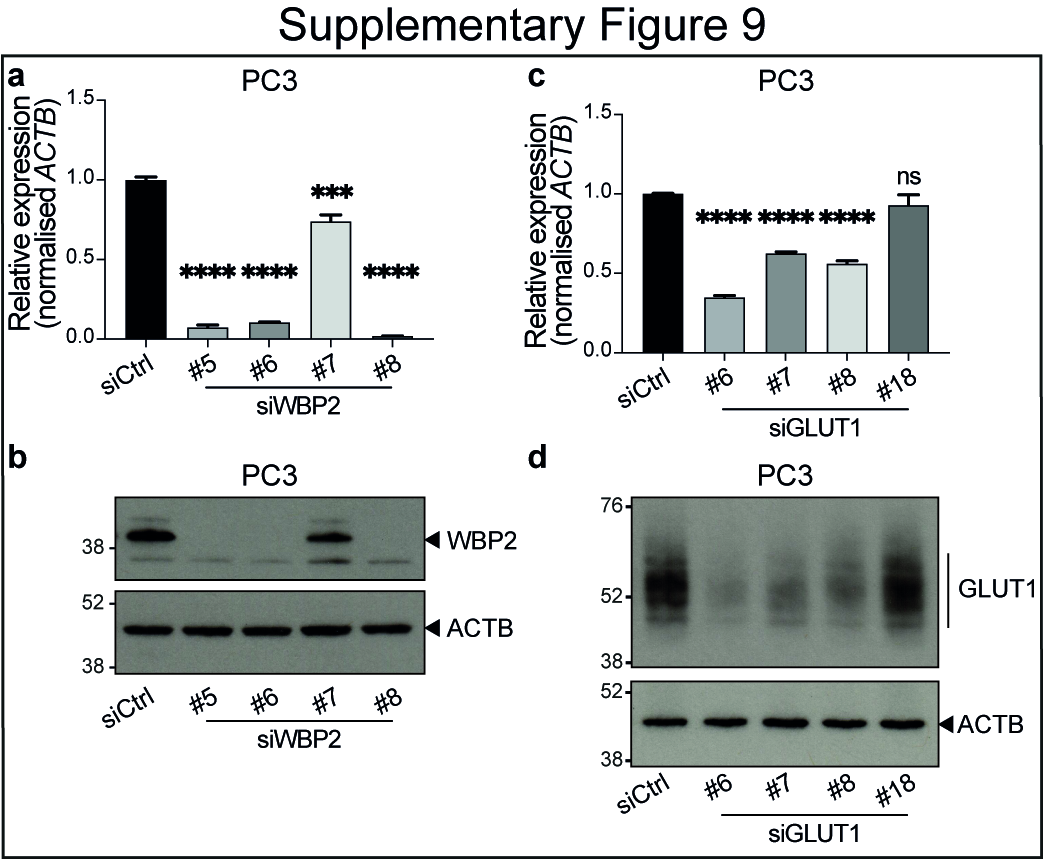

Supplement: Supplementary file 10 — Supplementary Figure 9 [file 41388_2022_2178_MOESM10_ESM.tif]

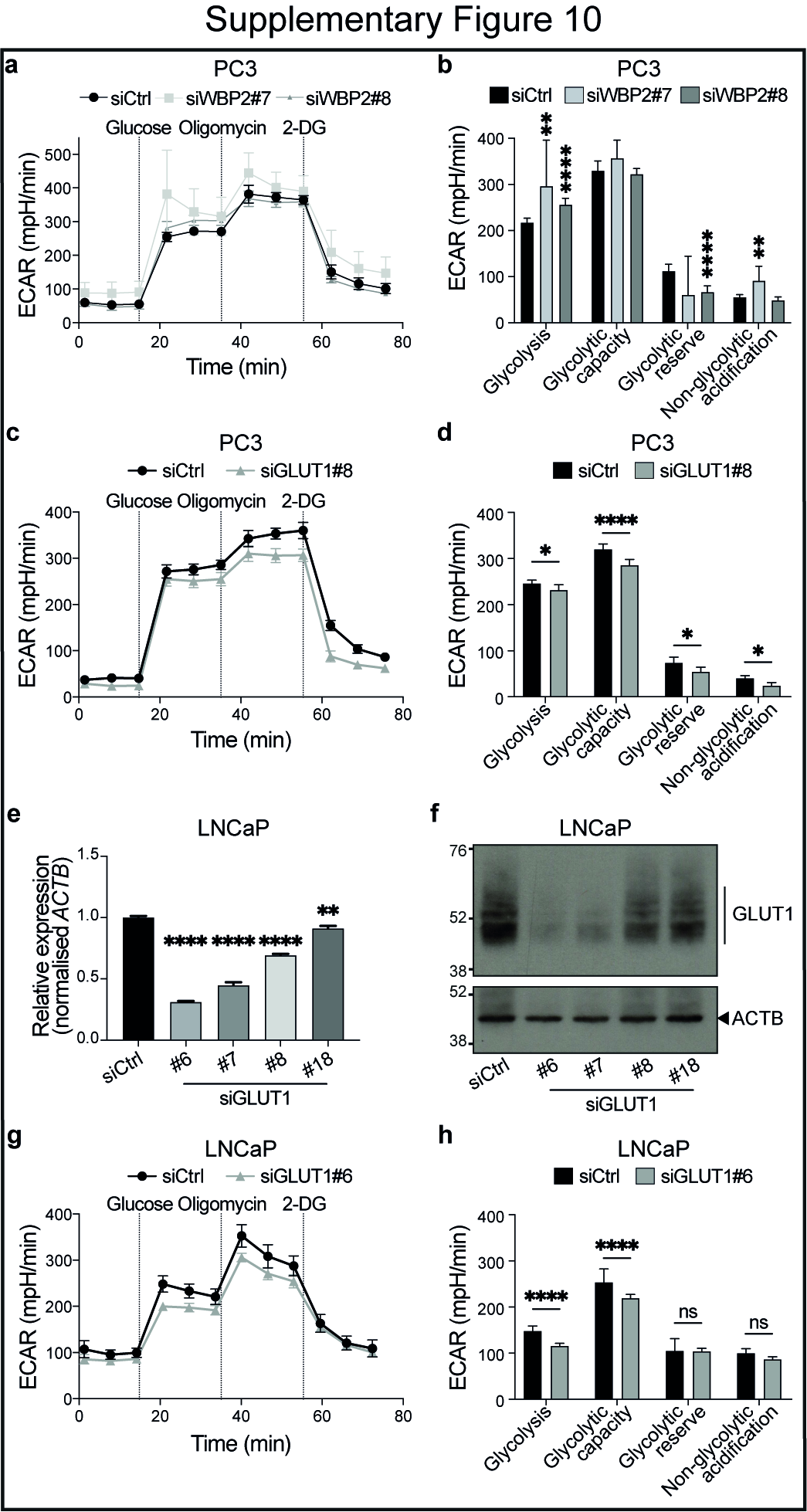

Supplement: Supplementary file 11 — Supplementary Figure 10 [file 41388_2022_2178_MOESM11_ESM.tif]

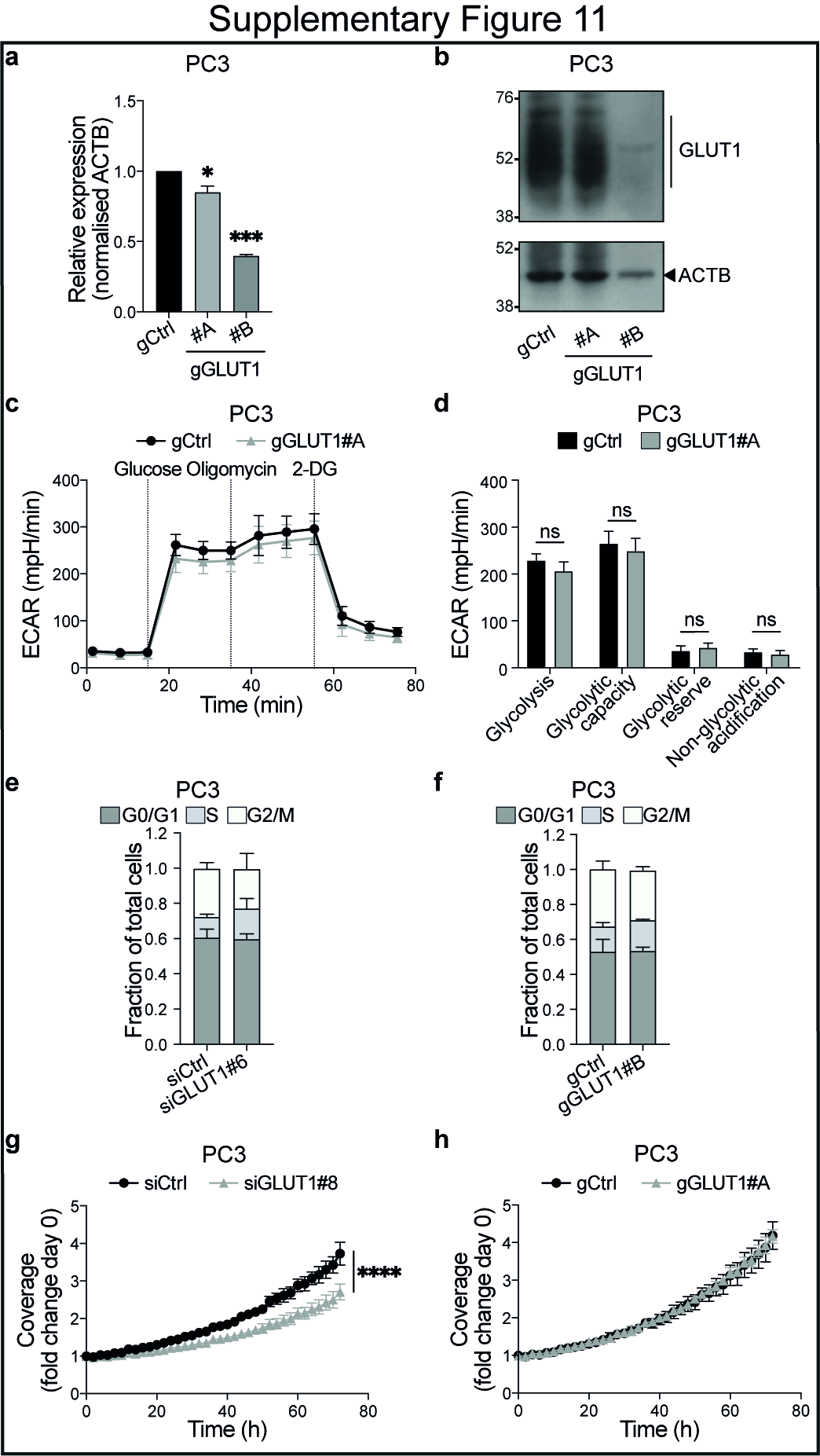

Supplement: Supplementary file 12 — Supplementary Figure 11 [file 41388_2022_2178_MOESM12_ESM.tif]
